# Subthalamic nucleus input-output dynamics are correlated with Parkinson’s burden and treatment efficacy

**DOI:** 10.1038/s41531-024-00737-8

**Published:** 2024-06-15

**Authors:** Xiaowei Liu, Jing Guang, Stefanie Glowinsky, Hodaya Abadi, David Arkadir, Eduard Linetsky, Muneer Abu Snineh, Juan F. León, Zvi Israel, Wei Wang, Hagai Bergman

**Affiliations:** 1 Department of Neurosurgery, West China Hospital, West China School of Medicine, Sichuan University, Guoxue Lane No. 37, Chengdu, 610041 Sichuan China; 2 The Edmond and Lily Safra Center for Brain Science, The Hebrew University, Jerusalem, Israel; 3 Department of Neurology, Hadassah University Hospital, Jerusalem, Israel; 4 Department of Neurosurgery, Hadassah University Hospital, Jerusalem, Israel; 5 Department of Medical Neurobiology, Institute of Medical Research Israel-Canada (IMRIC), The Hebrew University-Hadassah Medical School, Jerusalem, Israel

**Keywords:** Parkinson's disease, Neurophysiology, Parkinson's disease

## Abstract

The subthalamic nucleus (STN) is pivotal in basal ganglia function in health and disease. Micro-electrode recordings of >25,000 recording sites from 146 Parkinson’s patients undergoing deep brain stimulation (DBS) allowed differentiation between subthalamic input, represented by local field potential (LFP), and output, reflected in spike discharge rate (SPK). As with many natural systems, STN neuronal activity exhibits power-law dynamics characterized by the exponent α. We, therefore, dissected STN data into aperiodic and periodic components using the Fitting Oscillations & One Over F (FOOOF) tool. STN LFP showed significantly higher aperiodic exponents than SPK. Additionally, SPK beta oscillations demonstrated a downward frequency shift compared to LFP. Finally, the STN aperiodic and spiking parameters explained a significant fraction of the variance of the burden and treatment efficacy of Parkinson’s disease. The unique STN input-output dynamics may clarify its role in Parkinson’s physiology and can be utilized in closed-loop DBS therapy.

## Introduction

Beta oscillations in local field potentials (LFP) and spiking activity (SPK) in the subthalamic nucleus (STN) are considered the electrophysiological hallmark of Parkinson’s disease (PD)^1–6^. LFP recordings, performed within a week of electrode implantation, revealed many patients with peaks in low beta (LoBeta, 13–20 Hz), high beta (HiBeta, 20–35 Hz), and both beta sub-bands^4^. LoBeta oscillations are positively correlated with the severity of PD motor symptoms, and their power is suppressed by treatment with antiparkinsonian medication or deep brain stimulation (DBS)^1,4,5,7^. Chronic neuronal sensing and recording devices show beta activity as a consistent long-term biomarker for Parkinson’s symptoms^8–10^. These cumulative studies underscore that beta LFP activity can be considered a biomarker for PD’s motor symptoms.

Many centers utilize extracellular recording of spiking (action-potential) activity to assist in navigating to target brain regions during DBS surgery^11,12^. The SPK can serve as a representation of the output from the recorded neurons. In addition to SPK, LFP, specifically encompassing low frequencies (e.g., from 0.1 to 70 Hz), could be recorded in the brain’s extracellular space. LFPs are probably generated by subthreshold (e.g., synaptic activity) modulation of the membrane potentials^13^ and, therefore, can be used as a proxy for the input of the recorded structure. The exact relationship of LFPs to the SPK in the STN of PD patients is still unclear. Significant coherence was found between the LFP and SPK in the subthalamic region^3^. Our group reported similar results in the MPTP non-human primate model of PD^14^. However, a recent study reported that STN periodic spike bursts commonly preceded the LFP beta oscillation (i.e., the periodic bursts occur at the ascending phase of the LFP beta oscillation) and that other neuronal firing activity had no relationship to the LFP^15^.

Neural oscillations have been extensively studied using advanced methods in the time and frequency domains^16–18^. The traditional oscillation bands are predefined based on the canonical frequency bands and extracted by applying narrowband filtering. Typically, the power of a frequency band is either the maximum or the total power within that specific band. However, most physical and physiological phenomena follow a power law (1/f^α^, f represents the frequency, α is the exponent, or the slope of the power-frequency relationship in a log-log plot) rule^19^. The power at each frequency band is a summation of the aperiodic (1/f^α^) and periodic components^20,21^. Extracting the periodic oscillations and aperiodic components from the signals of interest by Fitting Oscillations and One Over F (FOOOF) analysis can overcome the limitation of traditional narrowband analyses^20,22^.

This study investigated the relationship and differences between LFP and SPK in the STN of PD patients undergoing DBS procedures. Utilizing the FOOOF algorithm^20^, we dissected these signals into periodic and aperiodic components to better understand their features. Our analysis also extended to examining the potential of these newly characterized STN physiological features as indicators for PD severity and for the predictions of the effectiveness of treatments.

## Results

This study was conducted on PD patients underwent DBS implantation in the STN during 2016–2021 at Hadassah Medical Center in Jerusalem, Israel (Demographic and clinical details are described in Supplementary Table 1). Electrophysiological recording of the STN activity and neighboring structures was done as part of the standard-of-care DBS navigation procedures.

All signals were recorded when the patients were awake and in a state of rest. The LFP and SPK were obtained by offline filtering the raw data at 3–200 Hz and 300–6000 Hz, respectively, using four-pole Butterworth zero-phase band-pass filters. The SPK was rectified^1^ to reveal the low-frequency (<300 Hz) oscillations in discharge rate (SPK, Supplementary Figs. 1, 2).

Based on our inclusion criteria, we included 308 out of 492 trajectories from 146 patients and 25,822 and 27,130 recording sites of LFP and SPK, respectively. We compared three subthalamic regions: the subregion preceding the STN, namely the internal capsule (Pre-STN), and the motor and non-motor domains of the STN, previously identified as the dorsal lateral oscillatory region (DLOR) and the ventral medial non-oscillatory region (VMNR)^1,23,24^. Further details are shown in Supplementary Table 1.

The FOOOF algorithm^20^ decomposed the neuronal activity into aperiodic and periodic components (Supplementary Fig. 1). The aperiodic exponents were used to whiten the power spectral densities (PSDs) of the LFP and the SPK activity for further analysis of the STN periodic components.

### The goodness of fit of the FOOOF analysis to the LFP and SPK activity

**Fig. 1:**
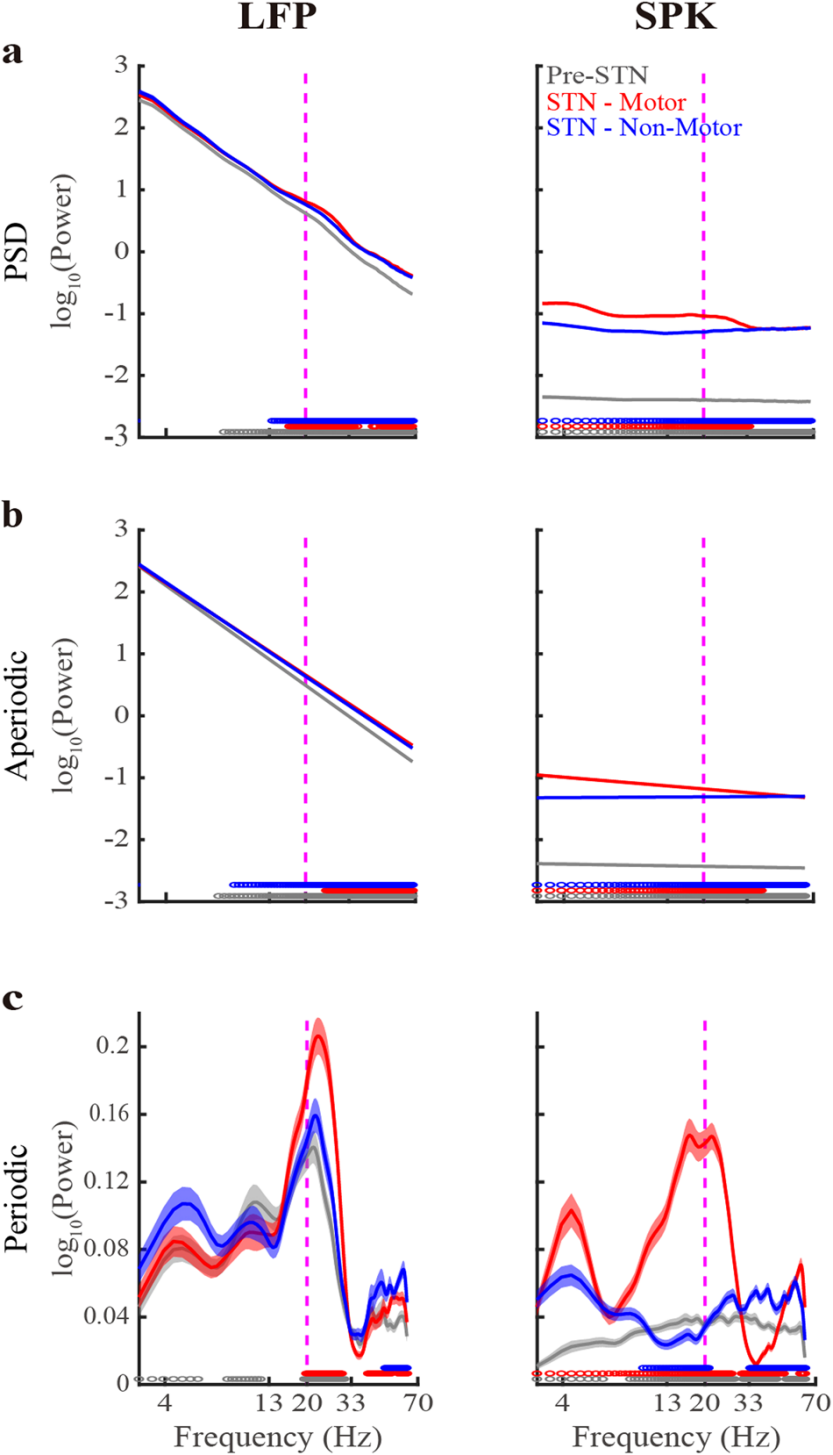
Robust differences in aperiodic and periodic components of subthalamic LFP and spiking (SPK) population activity. Left. The population average LFP Power Spectral Density (PSD, **a**) and its aperiodic (**b**) and periodic components (**c**) in three STN sub-regions. Right, same as the left subplot, but for the spiking (SPK) activity. Gray/red/blue lines indicate the pre-STN and STN motor and non-motor domains, respectively. Their corresponding shade lines indicate SEM. Colored circles above the x-axes represent the frequencies at which there was a significant difference between the pre-STN and STN motor domain (gray), between the STN motor and non-motor domains (red), and between the STN non-motor domain and pre-STN (blue). Significance was calculated using the Wilcoxon rank sum test and the Bonferroni correction (*p* < 0.05/3 = 0.0167). Vertical dashed lines denote the 20 Hz frequency point. See also Supplementary Figs. 3–7.

Figure 1 depicts the STN LFP and SPK population mean of the raw PSDs and their aperiodic and periodic components. The goodness of fit of the FOOOF analysis is assessed by the *R*^2^ and the mean absolute error (MAE, error) values. Optimally, *R*^2^ and error should be as close as possible to 1 and zero, respectively. The *R*^2^ values of LFP are 0.99 ± 0.01 (mean ± SD) in the three STN subregions. The *R*^2^ values of SPK are 0.64 ± 0.17, 0.89 ± 0.15, and 0.65 ± 0.17 in Pre-STN and motor and non-motor STN domains, respectively.

**Fig. 2:**
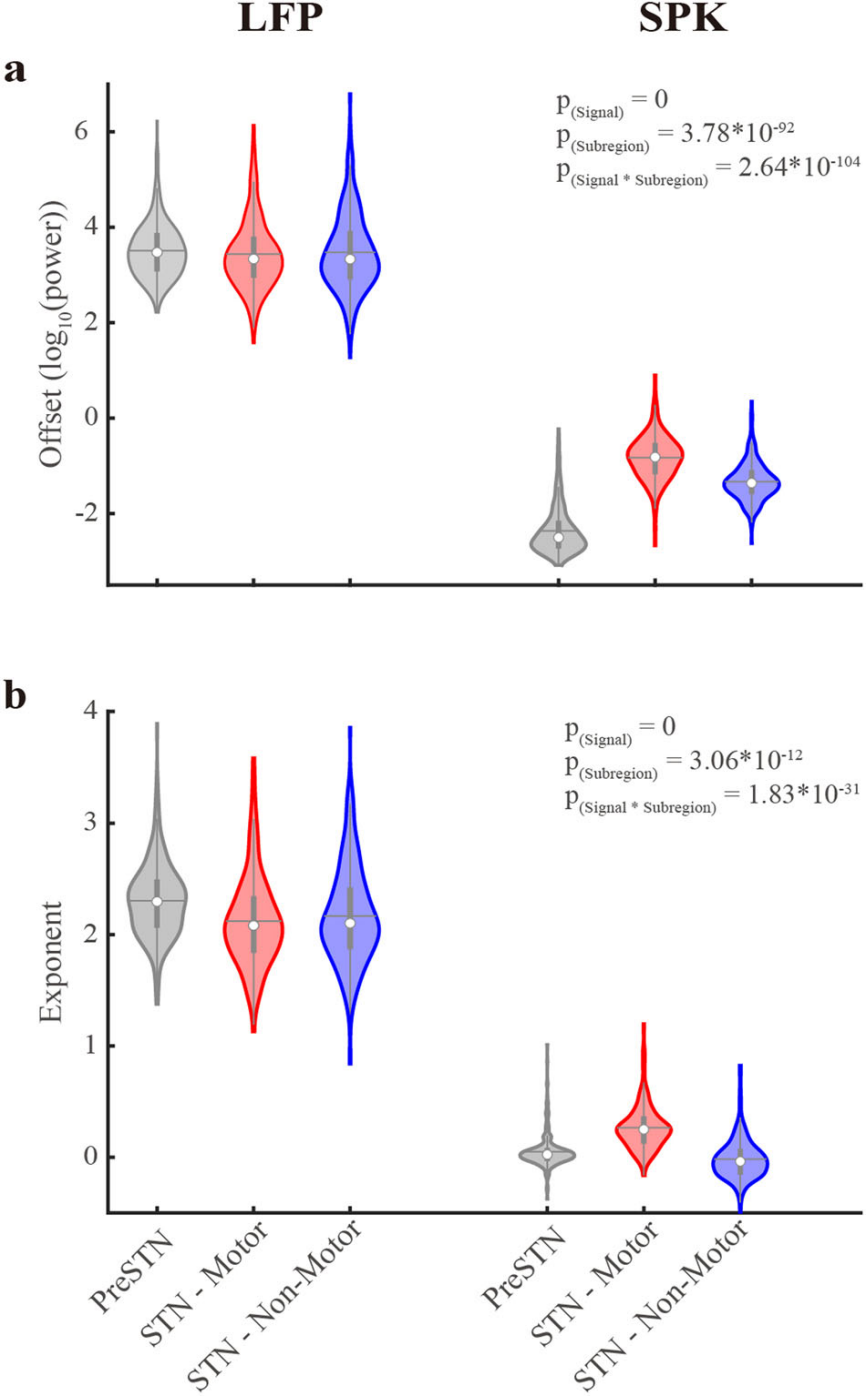
Significant differences in aperiodic parameters of LFP and spiking (SPK) activity in the subregions of the subthalamic nucleus. **a** The aperiodic offset parameter of LFP and SPK in three STN sub-regions. **b** As (**a**), but for the aperiodic exponent parameter. The pre-STN and the STN motor and non-motor domains are shown in gray, red, and blue, respectively. The contour of the violin plots shows the distribution of the data. The white circle shows the median. The horizontal gray line represents the mean. The gray vertical bold lines span from the 25th to the 75th percentiles of the sample, and the length of this line is the interquartile range. The lowest and highest whiskers of the violin plots are values that are 1.5 times the interquartile range below the 25th percentile and above the 75th percentile. The N-way analysis of variance was used to analyze the difference in aperiodic parameters. The *P*_(Signal)_, *P*_(Subregion)_, and *P*_(Signal*Subregion)_ represent the statistical probability for a significant difference in offset or exponent values between LFP and spiking (SPK) activity, for a difference of offset or exponent values between the three STN subregions, and for the interaction effect of offset and exponent values between the signal types and sub-regions. Detailed results of multiple comparisons are shown in Supplementary Table 2. See also Supplementary Figs. 3–7.

**Fig. 3:**
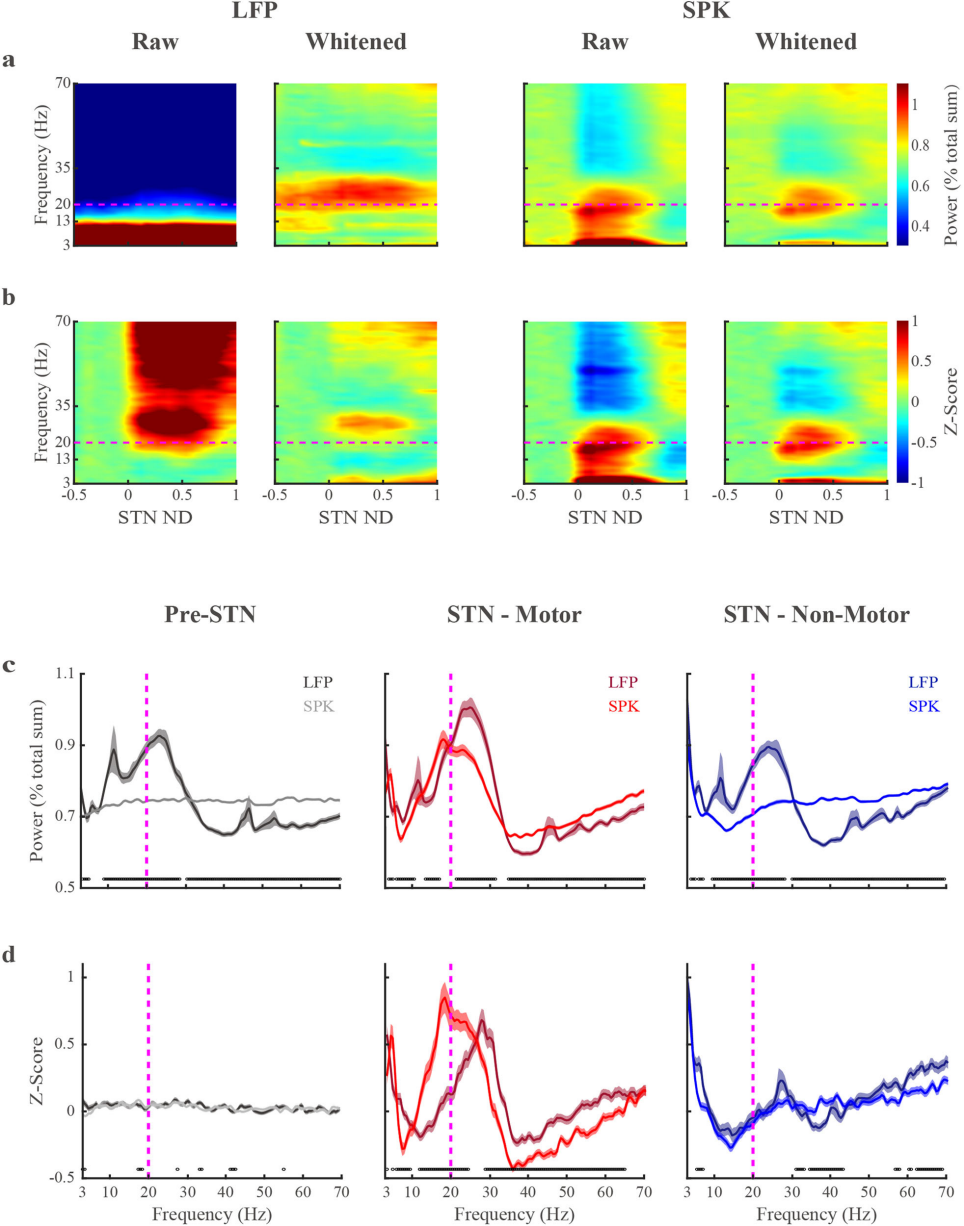
Raw and whitened averaged spectrograms and power-spectrum densities (PSD) reveal differences in beta frequency distribution and peak beta oscillations between LFP and spiking (SPK) activity in the motor domain of the subthalamic nucleus. **a** Raw and whitened spectrograms of LFP and SPK are normalized by the total amount of power in the tested frequency range (3–70 Hz) for each tested recording site (normalization by frequency). **b** The raw and whitened spectrograms are normalized by frequency (as in (**a**)) and by the power in the pre-STN domain per each frequency bin (normalization by distance). The spectrograms in the second and fourth columns of (**a**, **b**) are whitened in the frequency domain (Eq. (2), pwelch-FOOOF-whitening). The x-axis is the normalized distance (ND, normalized STN length from entry to exit equals 1). The entrance and exit of STN are represented by 0 and 1, respectively. The negative values on the x-axis indicate the pre-STN region. The y-axis is the frequency on a linear scale. The color scale of the power spectral density normalized by frequency (**a**) indicates the percentage power of the frequency bin out of total power. The color scale of the power spectral density normalized by frequency and distance (**b**) represents the deviation from the mean value of the first ten depths in pre-STN (z-score, standard deviation unit). **c** The PSDs of LFP and SPK are normalized by frequency in three sub-regions. **d** As in (**c**) but normalized by frequency and by distance (Pre-STN activity). The dark and light lines indicate the LFP and SPK in the pre-STN (gray) and the STN motor (red) and non-motor (blue) domains, respectively. Their corresponding shade lines indicate SEM. The black circles above the X-axes indicate frequencies at which there were significant differences (Wilcoxon rank sum test) between LFP and spiking activity. The horizontal (**a**, **b**) and vertical (**c**, **d**) magenta dashed lines are the referenced line of 20 Hz. See also Supplementary Fig. 8.

The lower *R*^2^ values in SPK can be attributed to its relatively lower exponent values compared to LFP (Figs. 1, 2b). Our numerical simulations (Supplementary Fig. 3) demonstrate that exponents nearing zero (as for our SPK activity) result in reduced *R*^2^ values. Moreover, the *R*^2^ value tends to grow as the absolute value of the exponent increases. The introduction of periodic components diminishes the exponent’s impact on *R*^2^ values. This explains why the *R*^2^ values of SPK in the STN motor domain with more prominent periodic components (Figs. 1,3) are higher than those in the two other subregions. Significantly, the error remains unaffected across an extensive spectrum of periodic power and exponent (α) values. Indeed, both LFP and SPK have low error values in three STN subregions (error < 0.04). We concluded that the FOOOF analysis yielded a robust fit for our data, prompting us to proceed with a comparative analysis of the aperiodic and periodic components within both STN LFP and SPK activity.

### Significant differences in aperiodic parameters between LFP and SPK activity

The aperiodic parameters are offset and exponent^20^. LFP exhibits significantly larger offsets than SPK in the three subregions (Figs. 1, 2a). There is no significant difference in LFP offsets between subregions, while SPK offsets in each subregion are similar but significantly different (Supplementary Table 2).

Additionally, the exponents of LFP and SPK also differ significantly (2.20 ± 0.40 and 0.11 ± 0.22, respectively, Fig. 2b). The exponents of LFP and SPK resemble those of Brown noise (α = 2) and White noise (α = 0), respectively. LFP exponent in the pre-STN is significantly larger than that in the two subthalamic regions (Supplementary Table 2).

We also examined the correlation between the aperiodic parameters of STN neuronal activity (Supplementary Fig. 4). A robust and significant positive correlation exists between the exponent and offset parameters within a signal type (i.e., LFP or SPK) in all recording areas. The correlation is strongest in the STN motor domain. Finally, recent clinical studies have increasingly adopted bi-polar differential recording techniques, frequently presenting LFP PSDs that deviate from the expected 1/f^2^ behavior^7–9^. Several factors contribute to this divergence, including inherent patient variability^25^, variations in impedance mismatch among lead contacts^26^, and the potential implementation of high-pass filters in certain investigations. Moreover, the spatial correlation of LFP aperiodic parameters, can result in a flattened PSD pattern after the application of differential operations in bipolar recordings.

### Significant differences in periodic (oscillatory) components between LFP and SPK activity

The bottom subplots of Fig. 1 depict the FOOOF-derived population average periodic activity of LFP and SPK in three STN subregions. Beta oscillations in LFP are observed in the three subregions, while in SPK, they only exist in the motor subdomain. LFP HiBeta (20–33 Hz) oscillations in the motor STN are significantly higher than in the two other subregions. There is no significant difference in LFP LoBeta oscillations between the three subregions. The Pre-STN usually points to the internal capsule (white matter). The LFP in Pre-STN probably represents the volume conductance originating from the cortex, the globus pallidus, and the STN^27–30^. The LFP in STN may also be affected by these structures, as well as by the LFP generated by neurons in STN^27–30^. Nevertheless, most of the volume conductance is probably from the cortex, because of the cellular and gross anatomy (spherical shell around the subcortical structures)^30^. These may be why the beta oscillations appear in the monopolar LFP raw recording in the three STN subregions.

SPK has a reduced beta power than LFP. Additionally, the frequency distribution of beta oscillations in SPK is shifted to the left relative to the LFP. LFP also shows theta and alpha oscillations in the three subregions. Still, SPK demonstrates theta oscillations only in the STN motor domain, and no robust oscillations are found in the Pre-STN and the STN non-motor domain.

### The efficacy of the analysis techniques and the reliability of the physiological phenomena

To ensure that the distinctions in aperiodic and periodic components between LFP and SPK are not attributable to artifacts stemming from the rectification of the SPK signal (Supplementary Fig. 1c), we additionally subjected the LFP signal to rectification for validation (Supplementary Fig. 5, and Supplementary Tables 3, 4). The rectified LFP has robust goodness of fit of FOOOF analysis (*R*^2^ ~ 0.99, error < 0.05). The aperiodic parameters of non-rectified and rectified LFPs don’t reveal significant qualitative differences. No significant qualitative differences exist between non-rectified and rectified LFP PSDs at the beta frequency range (>13 Hz). However, rectified LFP has higher periodic power in theta and alpha frequency bands than non-rectified LFP. This is in line with previous studies demonstrating that full-wave rectification of EMG demodulates and enhances underlying low-frequency components of the signal (“carrying” frequencies), which may not be observed in the original signal due to the greater power of higher-frequency components of the signal^31^.

We used a numerical simulation to verify further that our observed shift in the center frequency of beta oscillations in SPK compared to LFP is not due to our data processing methods. We simulated Brown noise signals to which we added beta modulation and spikes (Supplementary Fig. 6). The simulation demonstrates that LFP rectification smooths the power distribution in the beta region but doesn’t change the center frequency. After adding Poisson-distributed spikes following a threshold crossing, the offset and the exponent of the simulated LFP don’t change. Band-pass (300–2000 Hz) filtering of the wide-band signal leads to the loss of the low-frequency components. However, full-wave rectification reinstated the low-frequency (20 Hz) oscillatory component. These results reveal, in line with our previous studies^32^, that spikes don’t affect the LFP behavior and that rectification (absolute operator) of the spiking (>300 Hz) activity exposes the behavior of the discharge rate of the spikes.

Supplementary Fig. 7 shows the differences between the envelope of the discharge rate (SPK, as used in the DBS physiological navigation algorithms^33,34^ and this study) versus the analog broadband (3–9000 Hz) neuronal activity that includes both the LFP and the extracellularly recorded raw SPK. The broad-band neuronal signal can be well represented by power law distribution with exponent values higher than 2. However, such broad-band presentation of the neural activity masks the low-frequency oscillations that characterize the LFP and the discharge rate of the STN in the Parkinsonian state. This underscores the importance of analyzing the LFP and SPK signals separately, as is done in the remainder of this paper.

### Whitened and Z-normalized spectrograms reveal a highly distinctive and stable distribution of LFP and SPK beta oscillations in the STN motor domain

When neuronal activity is recorded over several recording sites, we can use spectrograms to represent the activity as a function of the distance to the estimated location of the target^1^ or to average the spectrogram over the distance to get the average power spectrum density. To overcome the confounding effect of the power-law behavior of the neuronal data, we used the FOOOF exponents to whiten the signals (in frequency and time domains) at the level of each recording site (Supplementary Figs. 1, 2). The average population spectrograms in Fig. 3a are shown before (raw, i.e., classical spectral analysis) and after whitening in the frequency domain. Unlike the raw spectrogram, the whitened LFP spectrogram demonstrates clear beta oscillations. However, the beta oscillations in the pre-STN suggest that LFP STN beta oscillations are confounded by volume conductance (the major source is probably from cortical activity)^27^. Robust intrinsic LFP HiBeta periodic activity in the STN may become visible after the removal of volume conductance activity. We used z-score normalization based on the pre-STN activity for each frequency bin to minimize the volume conductance effects (Fig. 3b).

In sharp contrast with the LFP, the raw SPK spectrograms are less affected by volume conductance. Notably, the whitened SPK spectrogram displays both LoBeta and HiBeta oscillations in the STN motor domain (Fig. 3a, b). Additionally, the whitened spectrograms were stable for each STN subdomain, and suggest that LFP and SPK have different distributions of beta oscillations in the STN motor subregion. This is also verified by whitening in the time domain (Supplementary Fig. 8).

### Lower peak beta frequency in SPK relative to LFP in the average PSDs of the STN motor domain

Following the demonstration of stable (over distance in each STN subregion) periodic components in the SPK and LFP spectrograms (Fig. 3a, b), we moved to quantitative analysis of the average (population) whitened PSDs in the three STN subregions (Fig. 3c). In the STN motor domain, whitened LFP and SPK have clear beta oscillations. Whitened LFP has peak beta power in HiBeta, while whitened SPK is in the LoBeta range.

SPK beta oscillations were detected only in the STN motor domain, whereas LFP beta oscillations appeared in all STN subregions. We, therefore, applied z-score normalization based on the pre-STN activity to reduce the confounding effects of volume conductance on the STN LFP activity (Fig. 3d). The Z-normalization minimized the beta oscillations outside of the STN domain. The locations of the peak beta power in whitened LFP and whitened SPK are still above and below the 20 Hz (HiBeta-LoBeta dividing line), respectively. The beta oscillations in whitened LFP in the STN non-motor domain may be influenced by several factors. One potential source is the STN’s motor domain, where the crossover of oscillations could stem from the imprecise demarcation of the boundaries between STN subregions. This lack of precision in distinguishing the motor and non-motor transition zones within the STN could be due to the limitations of the current subregional detection algorithms, coupled with the anatomical nuances of these gradually transitioning subregions^29,34^. Volume conduction from the STN’s motor domain and pre-STN may also contribute to the beta oscillation in the STN’s non-motor domain.

**Fig. 4:**
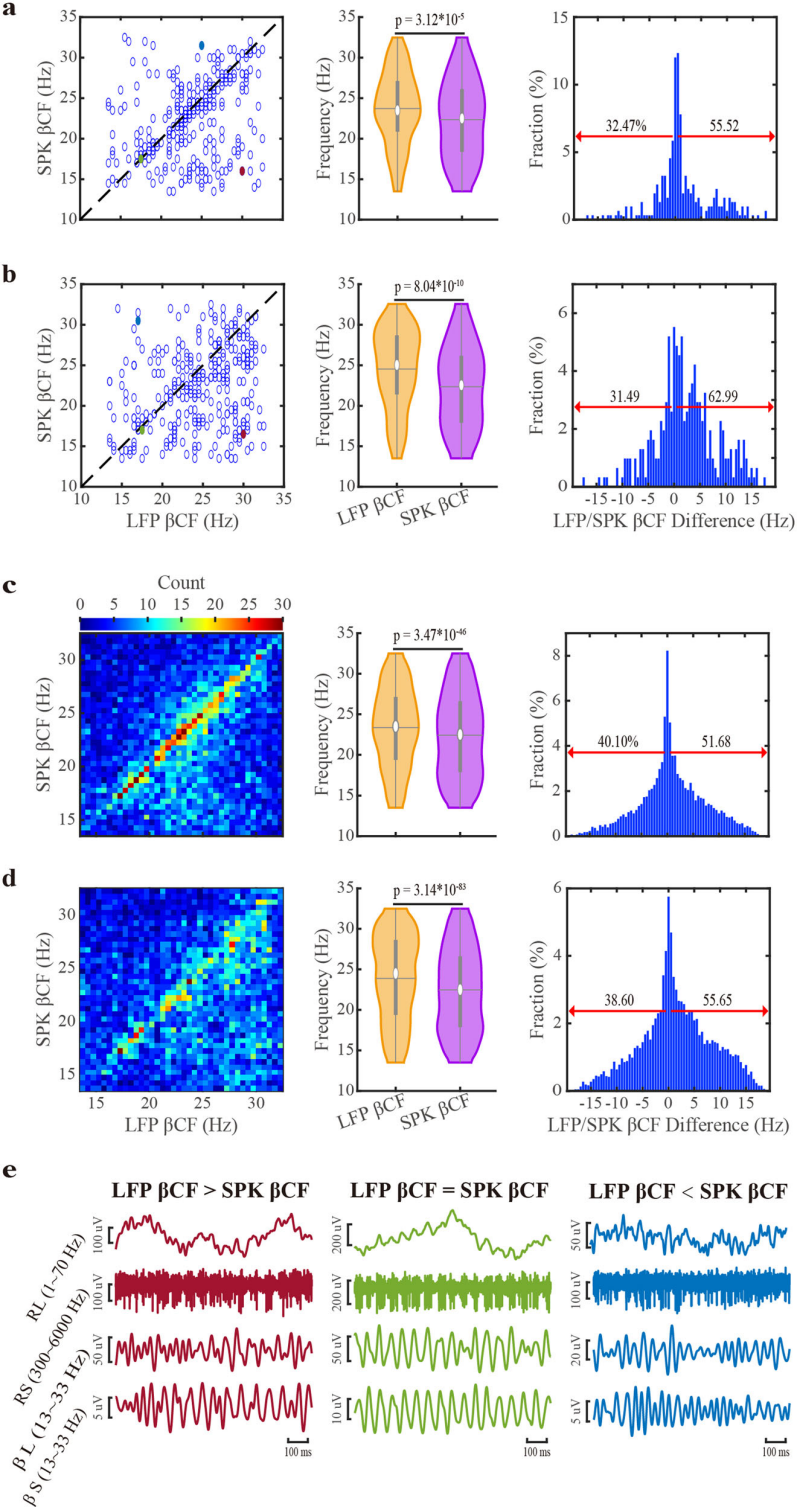
Downshift of the center frequency of beta oscillations of spiking (SPK) activity compared to LFP within the motor subdomain of the subthalamic nucleus. **a**, **b** Raster display, violin distribution, and the fraction of down- and up-shift of LFP and SPK center frequency of beta oscillations (βCFs) of STN trajectories. **c**, **d** As in (**a**, **b**), but the unit of βCFs is a single recording site. The βCFs are obtained from the frequency-normalized power spectra in the (**a**, **c**) subplots and from the frequency- and distance-normalized power spectra in the (**b**, **d**) subplots. The dark dashed lines on the left panel of (**a**, **b**) are the diagonal lines (at which *x* *=* *y*). The violins in the middle panel of (**a**, **b**, **c**, **d**) demonstrate the distribution of βCFs of LFP and SPK. The significance levels shown in the violin plots were calculated using the Wilcoxon signed rank test. In the right panel, the red arrows indicate the percentage of SPK βCFs that were upshifted (left) and downshifted (right) compared to the corresponding LFP βCFs. **e** shows the raw signals of three examples (LFP βCF is larger than, equal to, or smaller than SPK βCF, from left to right). The examples shown in (**e**) are marked in the corresponding colors (red, green, and blue) on the left panel of (**a**, **b**). RL raw LFP, RS raw SPK, βL β frequency band of LFP, βS β frequency band of SPK. See also Supplementary Figs. 9, 10.

The frequencies of beta oscillations are different across patients. However, they are stable along a single STN trajectory and for different trajectories of the same patients^1^. To further explore the relationship between the frequencies of whitened LFP and whitened SPK beta oscillations, we calculated their beta center frequencies (βCFs) for the STN motor subregion of each trajectory (Fig. 4a). The raster displays of the same trajectory LFP and SPK βCFs reveal a robust tendency towards the right-lower half (LFP *β*CF > SPK *β*CF). LFP *β*CF is significantly higher than SPK βCF, and there is a larger fraction of pairs whose LFP βCF is more prominent than their corresponding SPK *β*CF. We also estimated *β*CF in PSDs normalized by frequency and distance (i.e., Z-normalization by the pre-STN activity). The SPK *β*CF is relatively downshifted more, and the percentage of sites with LFP βCF larger than SPK *β*CF increases after the z-score normalization (Fig. 4b).

Figure 4c, d shows *β*CFs from simultaneously recorded LFP and SPK signals of single sites in the STN motor domain calculated from the PSDs normalized by frequency and Z-score, respectively. At the level of the single recording site (*n* = 9147), SPK *β*CF also tends to shift downward relative to LFP *β*CF. Typical examples are shown at Fig. 4e. Whitening in the temporal domain yields similar results (Supplementary Fig. 9).

We have also calculated the regular and whitened magnitude-squared coherence (Supplementary Fig. 2) of the simultaneously recorded (in the same recording site) LFP and SPK to estimate their frequency overlap and synchronicity (Supplementary Fig. 10). The coherence in the beta frequency band is higher in the STN motor domain than in the other subregions. No significant difference exists between regular and whitened coherences in the two STN subregions. Thus, the distribution of LFP and spiking beta oscillations overlapped in the STN motor domain (in line with Fig. 3 and the data along the diagonal in Fig. 4a–d).

### The broader and asymmetric distribution of population SPK and LFP beta oscillations reflects a wide distribution of narrow and symmetrical frequency oscillations at single sites

**Fig. 5:**
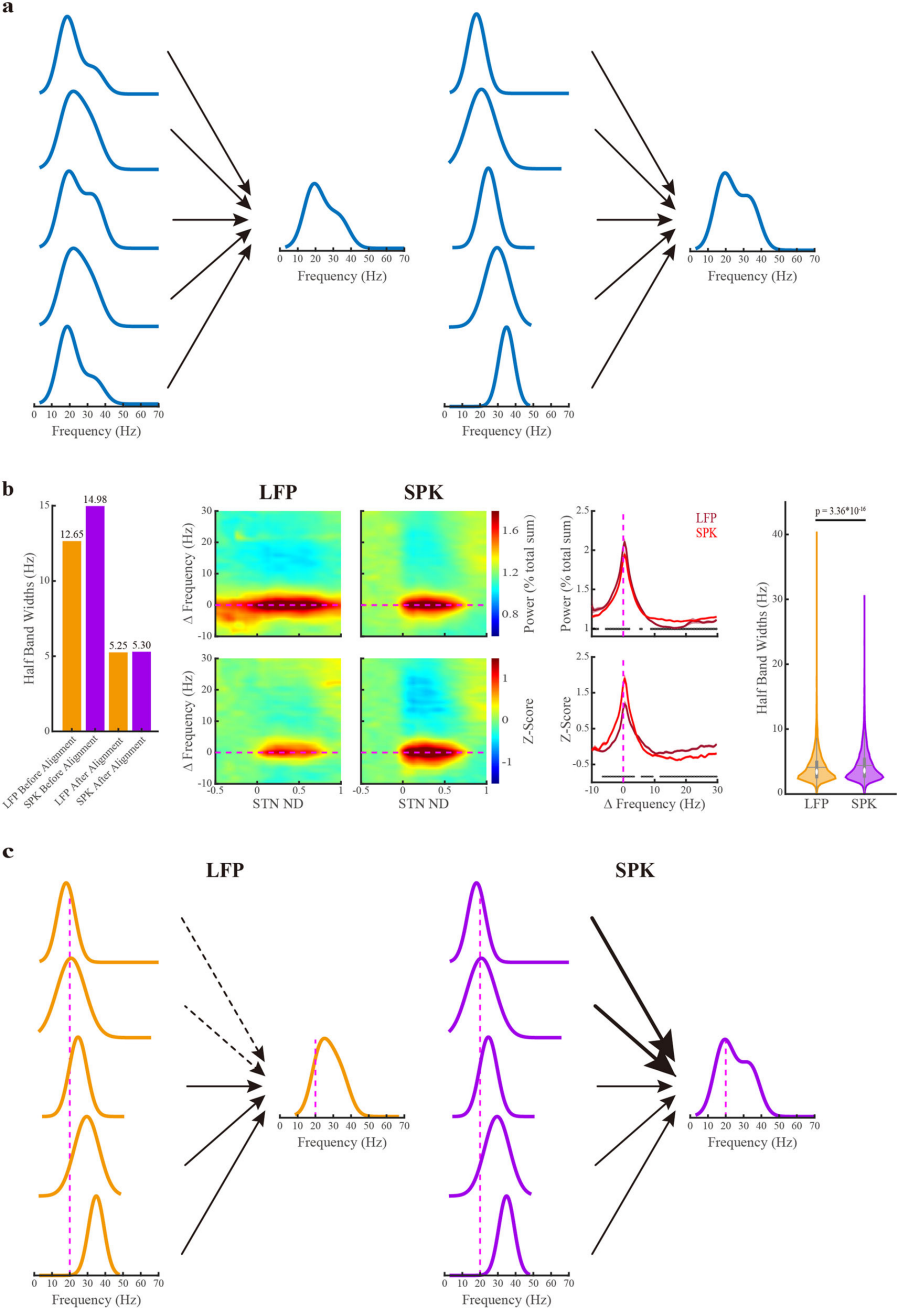
The distribution of beta oscillations of LFP and spiking (SPK) activity in a single site is narrower than the population distribution of beta oscillation in the STN DLOR. **a** The potential scenarios for generating population-wide power spectral density (PSD): the population broad and asymmetric PSD might be caused by the broad and asymmetric PSDs in single sites (the left panel) or the broad and asymmetric distribution in single sites with narrow and symmetric PSDs (on the right panel). **b** The population half-band width of LFP and SPK beta oscillations in the motor domain of the subthalamic nucleus. The first and second orange/purple bars indicate the half-band width of LFP/SPK before and after the alignment to the peak beta frequency, respectively. The LFP (left panel) and SPK (right panel) spectrograms are whitened in the frequency domain, and their frequencies are shifted to the peak beta frequency. The color scale in the first and second rows of the spectrograms indicates the percentage of total power and the standard deviation from the mean value of the first ten depths in pre-STN (z-score), respectively. The power spectrum densities are the averaged spectrum of LFP (dark red line) and SPK (light red line) in the STN motor domain. Their corresponding shade lines indicate SEM. The power spectrum is normalized by frequency (upper subplot) and by frequency and distance (lower subplot). On the right, the violin plots depict the distribution of half-band widths of LFP and spiking beta oscillations (4.10 ± 2.34 Hz vs 4.40 ± 2.62 Hz (mean ± SD), respectively) in each recording site. The Wilcoxon signed-rank test was used for pairwise comparison of half-band widths between LFP and spiking activity. **c** The potential mechanism for the downshift of beta center frequency in SPK (the right panel) relative to LFP (the left panel). The black dashed arrow lines (left panel) indicate a lesser impact, and the black bold arrow lines (right panel) represent a greater impact of single-site PSD on population PSD. The horizontal or vertical magenta dashed line is the reference line of the peak beta frequency (ΔFrequency = 0 Hz) in subplot (**b**), or the reference line of the 20 Hz in subplot (**c**). See also Supplementary Figs. 11, 12.

The broad and asymmetric distribution of LFP and SPK beta oscillations (Fig. 3) may reflect different scenarios. It could result from many single-site oscillations with similar wide and asymmetric PSD (Fig. 5a-left) or broad and asymmetric distribution of single sites with narrow and symmetric PSD (Fig. 5a-Right). The finding of the downshift between LFP and SPK *β*CFs (Fig. 4) is consistent with both scenarios. We, therefore, calculated the half-band widths and half-side widths at the half-height of the beta peaks in 9147 sites (from the STN motor domain of 308 trajectories), where both LFP and SPK beta oscillations were simultaneously detected (Fig. 5b and Supplementary Fig. 11).

In the raw power spectral densities, the population half-band widths of LFP are narrower than that of SPK. After aligning each PSD to its corresponding βCF, the population half-band widths of both LFP and SPK activities exhibit similar half-band widths. For both LFP and SPK, beta oscillations are symmetric in both aligned populations and single sites. Similar results were obtained for the 1/4 and 3/4 height bandwidths (Supplementary Figs. 11, 12). Thus, the downshift of beta oscillation frequency from LFP to SPK reflects a population downshift of narrow and symmetric SPK PSDs compared to LFP PSDs in the STN (Fig. 5c).

### Subthalamic aperiodic parameters and spiking activity explained a significant fraction of the variance of the burden of Parkinson’s disease and the efficacy of its treatment

**Fig. 6:**
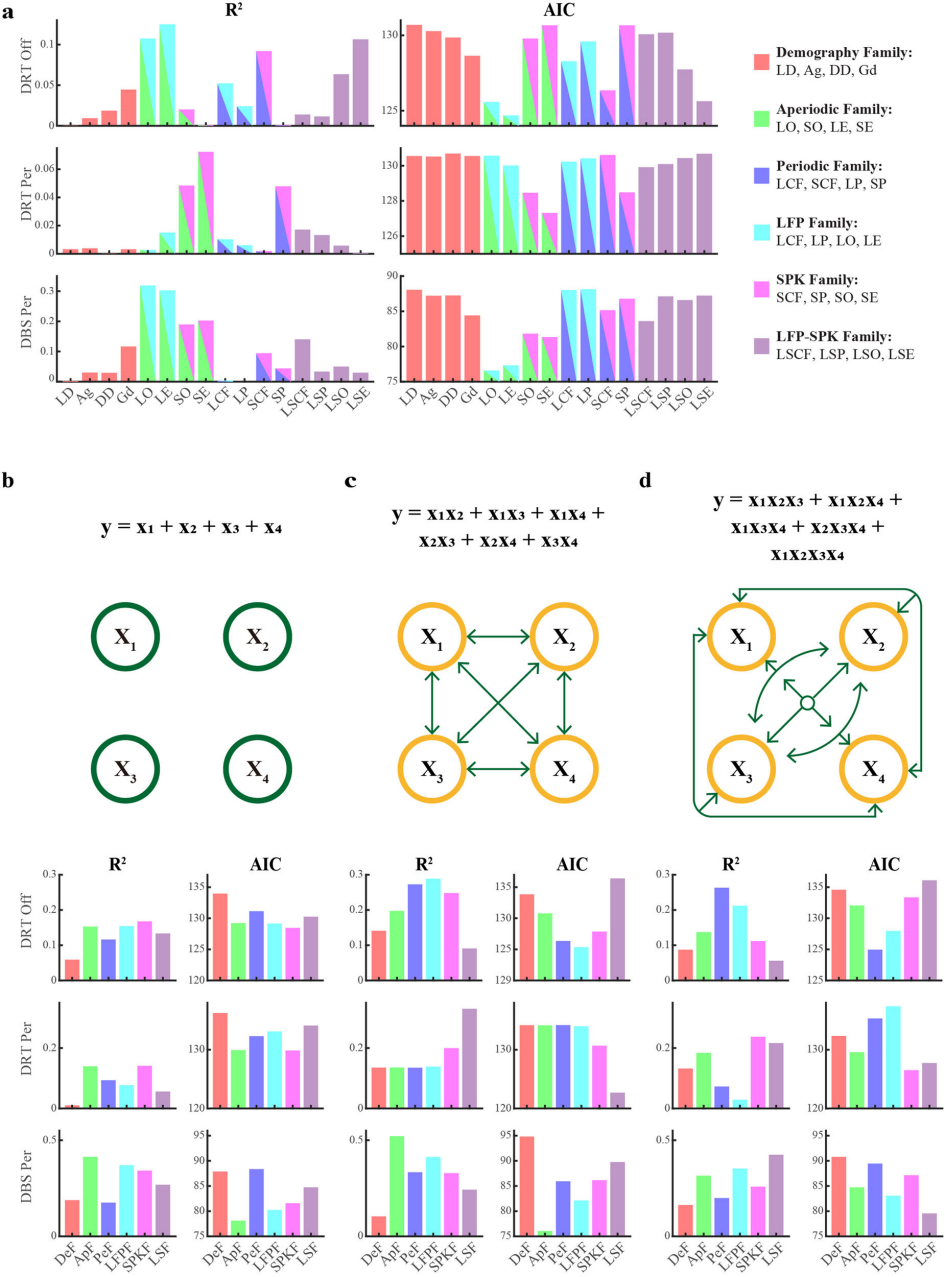
Evaluating predictive factors for pre- and post-operative clinical scores in patients with Parkinson’s disease. **a** The predictive relationship (*R*^2^) between individual predictors and responses (The pre-operative UPDRS III scores off dopamine replacement therapy (DRT Off), its percentage change due to pre-operative medication (DRT Per), and its percentage change due to DBS (DBS Per), and their corresponding Akaike Information Criterion (AIC, lower values indicate more probable fitting models). The individual predictors are levodopa daily equivalent dose (LD), age (Ag), disease duration (DD), gender (Gd), LFP beta center frequency (LCF), SPK beta center frequency (SCF), LFP beta peak power (LP), SPK beta peak power (SP), the difference between LCF and SCF (LSCF), LP and SP (LSP), LFP offset (LO), SPK offset (SO), LFP exponent (LE), SPK exponent (SE), the difference between LO and SO (LSO), and LE and SE (LSE). Predictor families are categorized as follows: Demography (DeF, red), Aperiodic (ApF, bright green), Periodic (PeF, blue), LFP (LFPF, cyan), SPK (SPKF, magenta), and the LFP-SPK differential (LSF, light purple). The elements of the predictor families are given in the top-right legend. **b** Goodness-of-fit (*R*^2^) and AIC for zeroth-order interaction of members in a predictor family. The color-coding matches (**a**). This represents the independent actions of the constitute members in a predictor family (like the formula and schematic diagram shown on the top of subplot (**b**). X_1_, X_2_, X_3,_ and X_4_ indicate the four members in a family). **c** *R*^2^ and AIC for first-order interaction of members in a predictor family. This general linear model integrates the interaction of each two members in a family, and their fitting formula and schematic diagram are demonstrated on the top of subplot (**c**). **d** *R*^2^ and AIC of higher-order general linear model fitting. The higher-order fitting indicates the integration of the interaction of each three members and the interaction of all (four) members in a family. The corresponding fitting formula and schematic diagram are demonstrated on the top of subplot (**d**). The predictor families in subplots (**c**, **d**) are identical to those in (**b**), and colors correspond to the predictor families in (**a**). See also Supplementary Figs. 13–16.

The dissection of the neural activity into aperiodic and periodic components has revealed new features of the neuronal activity in the STN motor subdomain of Parkinson’s patients. However, the relative contribution of these features to the burden and the clinical efficacy of dopamine replacement and DBS therapies have not yet been explored. To determine how well demographic and neuronal parameters, as well as various non-mutually exclusive parameter/predictor groups/families (including demography, aperiodic components, periodic components, LFP features, SPK features, and LFP-SPK comparisons), predict clinical symptoms and therapeutic effects in patients, we employed a battery of regression models. These regression models range from simple linear regression (Fig. 6a) to more complex general linear regression (general linear model (GLM) fitting) with various interaction orders (zeroth-order interaction of members in a parameter family is shown in Fig. 6b; first-order interaction of each two members in a parameter family in Fig. 6c; higher-order interaction of each three and four (all) members in a parameter family in Fig. 6d).

Both *R*^2^ and Akaike Information Criterion (AIC) were used to evaluate the goodness of GLM fitting. The *R*^2^ indicated the predictive capability and higher *R*^2^ values suggested a stronger predictive capability. The AIC manifested the model fitting and complexity. Lower AIC values indicated a more favorable balance between model fit and complexity. For patients with akinetic/rigid, the simple linear regression of each parameter (Fig. 6a) and zeroth-order interaction of parameter family (Fig. 6b) demonstrated that STN aperiodic and spiking parameters had a relatively better predictive capability of PD severity and therapy effectiveness. The predictive capability of DBS therapy from aperiodic parameter families was enhanced using the first-order interaction GLM fitting. This fitting model also decreased its AIC value (Fig. 6c). The first-order interaction GLM fitting increased the ability of aperiodic family predicting the PD severity but also increased the complexity of fitting (Fig. 6c). However, the contribution of these two parameter families was relatively weakened with the higher-order interaction GLM fitting (Fig. 6d). PD severity and DBS therapy had the strongest response to LFP family and aperiodic family in the first-order interaction GLM fitting, respectively (Fig. 6c). Our results also demonstrated that the demographic and neuronal parameters had a better ability to predict the DBS therapy than to predict the PD severity and the dopamine therapy in different level fitting (Fig. 6b–d). Roughly, the neuronal parameters were more effective to predict the burden and the treatment outcome of the disease than the demographic parameters (Fig. 6b–d).

The regression analysis yielded different results for patients with akinetic/rigid symptoms vs. tremor-dominant patients, for the different predicted variables (e.g., Parkinson’s severity, dopamine, and DBS efficacies), and for the various models (Supplementary Figs. 13–16). This is probably due to gaps in our data, intrinsic variability, and noise in our database. However, our results are in line with previous studies^6^. Nevertheless, the different regression models used here indicate a significant role for the aperiodic and SPK features and their interactions in predicting the Parkinson’s burden and the efficacy of dopamine replacement and DBS efficacy.

## Discussion

In this study, we highlighted the differences and relationship between LFP and the discharge rate (SPK) of STN neurons in Parkinson’s patients by separating STN neuronal activities into aperiodic and periodic components^20^. We found that the LFP exponent resembled Brown noise (α=2) whereas the SPK exponent is close to the White noise (*α* = 0). In the periodic components, we unexpectedly found that the beta center frequencies (βCFs) were downshifted in SPK relative to LPF in the STN motor region. This shift was not caused by a change in an asymmetric broadband distribution of neuronal beta oscillations. Instead, our results indicate a different distribution of symmetric, narrow-band oscillations of STN LFP and SPK activities. Finally, different regression models reveal that the new features of the STN neural activity (e.g., aperiodic components and LFP-SPK differences) are correlated with the Parkinson’s burden and the efficacy of dopamine replacement and DBS therapy.

We found that the LFP displays power-law behavior. The power is inversely and linearly related to the frequency in log-log plots, i.e., there is 1/f^α^ scaling of the power^21^. The exponent can be affected by many factors^13^, one of which is the relative contribution of excitation and inhibition (E/I ratio)^22,35^. However, detailed quantitative anatomy of the relative number and their somatic/dendritic location of STN synaptic input is still missing^36,37^. Additionally, the E/I balance reflects the physiological efficacy of the synaptic inputs, which is significantly affected by the frequency and pattern of discharge of the GPe^38^ and probably of cortico-STN neurons. Thus, our results showing different exponent values in pre-STN and STN domains cannot be easily framed with the suggested relationships with the E/I ratio. Our results could be explained by the degree of neuronal expenditure. Greater neural expenditure causes flatter slopes (smaller exponent)^39,40^. In PD, the activation of the basal ganglia is profoundly altered, and STN activity is significantly elevated^14^. We, therefore, expect the LFP exponent in STN to be smaller than that in Pre-STN. There are other possible explanations for our results, and future studies should explore the neuronal/metabolic correlates of the exponent to address this question.

The SPK of a neuron is a train of action potentials (spikes). To minimize the duration of the physiological navigation during the DBS procedure, we record the multi-unit activity of the STN. To do so, we filtered the raw signals with the 300–6000 Hz bandpass filter and then rectified the SPK by the absolute operator^32^, resulting in a SPK signal indicating the neuronal discharge rates of the total activity recorded by our electrodes. This differs from most previous studies that have used the SPK (e.g., 300–3000 Hz) of well-isolated single neurons^37,38^, however, at the price of masking low-frequency oscillations^31^.

Studies reported the aperiodic offset correlates with both neuronal population spiking and the related fMRI blood-oxygen-dependent signal^41,42^. In our current study, SPK offsets are significantly smaller than LFP offsets in the three subregions, and the SPK offsets are significantly larger in STN than in Pre-STN (Fig. 2a and Supplementary Table 2). Additionally, there is no significant difference in LFP offsets between subregions, while SPK offsets are significantly different (Fig. 2a and Supplementary Table 2). This is consistent with the previous studies. Because LFP is more likely the result of slow sub-threshold currents (primarily post-synaptic potential) of a large neuronal population within several millimeters, and SPK is usually considered as the action potentials emitted by nearby neurons^13^.

SPK exponents are around zero, which resembles the characteristics of a random process (white noise). This is in line with the Poisson-like distribution of SPK^43^, the tendency to a flat spectrum of cortical and pallidal units^44,45^, and the demonstration that the PSD of the aggregate of spike trains (with Poisson pattern and refectory period) has a flat spectrum, resembling that of white noise^46^. White noise background probably enables a better signal-to-noise ratio of the SPK responses to external or internal events. We, therefore, suggest that the background activity of the STN (as of many structures in the nervous system) is probably random to maximize the system’s information capacity and the signal-to-noise ratio of the evoked activity. In any case, the possible mechanism, biological significance, and application of the aperiodic parameters of STN SPK require further study, such as exploring their prediction of the STN subregions for better detection of DBS targets.

LFP more likely represents slow sub-threshold currents (primarily post-synaptic potentials) of a large neuronal population and is considered a proxy of the ‘input’ to the local neural network^13^. LFP beta oscillations in the STN of PD patients are possibly: (1) generated within STN through the network functional connectivity, i.e., driven by the STN afferent inputs^3,7,47,48^; (2) generated by the STN neurons themselves (intrinsic properties and subthreshold somatic activity)^15^; (3) generated by the volume conductance of LFP from other locations (such as the cortex)^28,30^. LFP and SPK beta oscillations aren’t always simultaneously present in the same recording electrode^28^. In addition, the amplitude of beta oscillations in LFP in the STN motor domain is higher than that of STN SPK oscillations^3,28^ (Figs. 1, 3). These results support the notion that mono-polar recorded STN LFP mainly results from afferent inputs and volume conductance. Notably, a major fraction of the volume conductance is from the cortex, which is also a significant source of STN afferents (the hyper-direct pathway). Thus, there is a significant overlap of the possible sources of STN LFP activity, and the STN LFP can be used as a proxy for STN input.

We can consider the STN SPK as reflecting the ‘output’ (since the fraction of interneurons in the basal ganglia structures is negligible)^32,49^. Parkinson’s pathologic mechanism may, therefore, be better understood by exploring the ‘input-output’ or LFP-SPK relationship in the STN. Previous studies indicate that in the STN of PD: (1) the firing of neurons is phase-locked to LFP beta oscillations^3,15^; (2) the power of LFP is coherent with that of SPK in the beta frequency band^50^; (3) the beta phase of LFP modulates the amplitude of the LFP high-frequency oscillations (HFO, 200–500 Hz). However, most of these studies were conducted on a small number of patients, and the aperiodic components of the STN activity might confound their analysis of periodic phenomena. Finally, these studies are in line with our finding of a sizeable fraction of neurons with similar frequency of LFP and SPK oscillations (trajectories/units close to the diagonal in Fig. 4 and Supplementary Fig. 9) and the LFP-SPK coherence (Supplementary Fig. 10).

Our study shed light on the STN input-output question, revealing a downshift of the βCFs of periodic oscillations from LFP (input) to SPK (output). This is correct even after z-score normalization to remove the volume conducted LFP activity. The downshifted βCFs between SPK and LFP suggest a non-linear input-output transformation of STN beta oscillations. The STN neurons encode and integrate their inputs from the cerebral cortex, thalamus, and GPe and then construct their outcome as SPK (i.e., information filtering). While LFP may be equally affected by all synaptic inputs, the SPK is more affected by excitatory synapses and synapses close to the soma. This is a possible source of the non-linearity that causes the βCF downshift toward the low-beta range in this input/output encoding/decoding STN process (Fig. 5c). That is, STN spikes can be dissociated from LFP^51^. Finally, we expect that the STN SPK, which drives the central and output structures of the basal ganglia, rather than LFP beta oscillations, to underly PD clinical symptoms.

The LFP and SPK in the STN of 146 PD patients were separated into periodic and aperiodic components using the FOOOF algorithm. We found the exponent of LFP resembled Brown noise, and the exponent of the discharge rate (SPK) was similar to white noise. We also found that the center frequency of beta periodic activity in the STN motor domain is downshifted in SPK compared to LFP. This downshift wasn’t caused by an asymmetrical and broad-band distribution of beta oscillations in a single STN recording site. Rather, it probably reflects the unique input-output relationships of STN neurons. Finally, the differences between the periodic and aperiodic components of the STN LFP and SPK subthalamic activity are correlated with the burden and the treatment efficacy of Parkinson’s disease. Future generations of DBS navigation and adaptive DBS algorithms may use these new features to optimize treatment in the spatial and temporal domains.

There are several caveats in our study worth noting. Firstly, this is a single-center study. Although the number of patients is bigger than in similar studies, our data still has limits and gaps. Secondly, the results were obtained from Parkinson’s patients undergoing DBS procedures. There are no signals from healthy individuals as a control group, or from Parkinson’s patients at early or late stages of the disease. Third, it’s difficult to distinguish between power law and log-normal behaviors based on our limited (one-two orders) frequencies (x-scale) tested. Other methods for estimating the exponent and enabling the detection of other features (e.g., knee) in the log-log plots were not tested here^52^. Finally, the frequency range tested started at 3 Hz, and lower frequency (Delta) oscillations were not included. However, the large number of patients and recording sites used in this study support the validity of the STN non-linear input-output relationship. This improved understanding of STN pathophysiology and the LFP-SPK beta downshift biomarker will likely pave the way for better adaptive DBS therapy. Future, much larger studies (with the number of patients in the range of thousands and more) should be done to verify the relationships between the neuronal parameters and the clinical symptoms and therapeutic efficacy. Overall, our results demonstrated that the neuronal parameters more effectively predict the outcome of DBS therapy than those of the demographic, including pre-operative dopamine treatment parameters^53,54^. Future studies should investigate whether this suggests distinct mechanisms of action between pharmacological treatments and DBS.

## Methods

### Patients

Patients with PD underwent DBS implantation in the STN during 2016–2021 at Hadassah Medical Center in Jerusalem, Israel (Supplementary Table 1). The patients had to be off medications starting the night before the DBS surgery. Inclusion criteria included clinically established PD, eligibility for DBS procedure, and available intraoperative electrophysiological data in the STN. Additionally, these patients consented to the operative procedure and signed informed consent. Patients were evaluated using the Unified Parkinson’s Disease Rating Scale (UPDRS) III both off and on medication before surgery, and on DBS postoperatively. In addition, the neurologist qualitatively evaluated the DBS outcome about 1 year after surgery, based on their subjective grading of the clinical status (+2: Excellent; +1: Very good; 0: Satisfactory; −1: Insufficient; −2: Poor). This retrospective study was approved by the Institutional Review Board (IRB) of the Hadassah Medical Organization, Jerusalem, Israel (0339-21-HMO). Patient consent for data use was waived by the IRB due to the retrospective nature of this study.

### Electrophysiological recordings

Data were acquired with NeuroOmega systems (Alpha Omega Engineering, Ziporit Industrial Zone, Nof HaGalil, Israel). In each hemisphere, two microelectrodes (Alpha Omega Engineering) were simultaneously inserted along the planned trajectory, targeting the STN in the central and posterior Ben Gun positions, 2 mm apart. In rare cases, only one microelectrode was used in the central position due to the patient’s anatomy indicating a higher risk of encountering blood vessels along the planned trajectory. All signals were recorded while the patients were awake, at rest, and off medications (overnight washout). The raw signal was sampled at 44 kHz and band-passed from 0.07 to 9000 Hz using a hardware 2 and 3-pole Butterworth filter, respectively. We began recording at 10 mm above the target, lowering the electrode 400 µm and 100 µm steps, before and in the STN areas, respectively. We recorded the neural activity for four seconds, after 2 s of stabilization, at each site until we exited the STN. Further details on microelectrode recordings and data acquisition can be found in our previous papers^33^.

### Trajectory selection

The length of STN and the subregional state sequence were detected based on the evaluative results of real-time recorded neuronal signals using the Hidden Markov Model (HMM). Finally, 308 trajectories were included for analysis out of a total of 492 microelectrode trajectories recorded during the relevant period (January 2016 - June 2021). The selection criteria included: (1) the chosen trajectory contained the pre-STN, and the STN motor and non-motor subregions; (2) each subregion was longer than one millimeter. The results reported here were similar when only the trajectories of the implanted leads were kept per hemisphere (n=225 trajectories).

### Signal pre-processing

The LFP and spiking discharge rate (SPK) signals were obtained by processing the raw data offline (Supplementary Fig. 1). The LFP was obtained by applying a zero-phase digital 4^th^ order band-pass Butterworth filter with cutoff frequencies 3–200 Hz (MATLAB R2020b) to the raw signal. We used a zero-phase digital 4th-order band-pass Butterworth filter of 300–6000 Hz to obtain the spiking signal. Following this step, we rectified the spiking signal by applying the absolute operator and then subtracted the mean (of the rectified signal) to get the SPK signal.

### Normalized root mean square (NRMS)

For each recording depth, the RMS of both the LFP and the SPK signals were calculated using Eq. (1)

1 $${x}_{{RMS}}=\sqrt{\frac{1}{N}\mathop{\sum }\limits_{n=1}^{N}{\left|{x}_{n}\right|}^{2}}$$

where *x*_*RMS*_ is the RMS value of this site, N is the number of samples in the time signal and *x*_*n*_ is the nth value of the time signal. We normalized the RMS values for each trajectory by dividing each RMS value by the average RMS value of the first ten sites (presumed to be an unbiased estimation of the baseline activity in the white matter).

### Outlier removal

If the value of RMS was more than 3 interquartile ranges above the upper quartile or below the lower quartile, the signal in this recording site was considered an outlier and was removed from analyses. The outliers were detected and excluded from the spiking and LFP signals based on their respective RMS.

### Power spectral density (PSD)

The PSD was estimated from both LFP and the SPK signals in each recording depth (site) using the Welch method (MATLAB R2020b’s pwelch function), with a Hamming window of 2 s (resulting in a frequency resolution of 0.5 Hz), 50% overlap, and frequency range from 3 to 70 or 200 Hz. Any site that was shorter in duration than 1.5 times the window size (i.e., <3 s) was excluded from the analysis. The PSD values of frequencies affected by the power-line noise (within 2 Hz of the 50 Hz frequency and its harmonics) were replaced by the mean value of the closest non-affected values. Substituting the values affected by the power-line noise by linear interpolating the closet values resulted in similar results.

The PSD was normalized either by frequency or by frequency and distance. For each recording depth, each PSD value was divided by the total power of the frequency range from 3 to 200 Hz to create a normalized by frequency PSD (NPSD). This normalization overcomes the effects of changes in total power (RMS) and reports the power per frequency as % of total power. Additionally, the mean value and the deviation of NPSD from the mean value of the first 10 depths in pre-STN were calculated for each frequency bin and used to normalize the LFP and SPK data by frequency and distance (Z-score).

### FOOOF analysis

We used the fitting oscillations & one over f (FOOOF) algorithm^20^ to separate neural power spectra into aperiodic and periodic components. We translated the FOOOF code from Python to MATLAB language. We added one fitting parameter (peak_width_limits_per) to avoid overfitting in high frequency. Each LFP and SPK PSD was fitted with the following settings: peak_width_limits = [0.8, 12], peak_width_limits_per = [0.02, 0], max_n_peaks = 6, min_peak_height = 0.05, peak_threshold = 2, aperiodic_mode = “fixed”. Aperiodic (offset and exponent) and periodic (center frequency, power, and bandwidth) features were extracted from the LFP and the SPK signals across the frequency range from 3 to 70 Hz. This frequency range was chosen to avoid the contamination of the low frequency artifact (<3 Hz) from heart rate, breathing or other sources, and to limit the inaccurate fitting in a broad range of frequencies.

The FOOOF aperiodic components of each recording site and signal type were used in the whitening procedures detailed below. Finally, we used Spearman’s Rho (correlation coefficient) to calculate the relationship between the aperiodic components (offset and exponent) of LFP and SPK in the three STN subregions. The correlation coefficient of the same aperiodic parameters pair of simultaneously recording sites (2 mm horizontal distance) was computed both before and after 100 shuffling iterations.

### Frequency domain whitening procedures

In each recording site, the PSD values from 3 to 70 Hz were whitened by multiplying each power by its frequency to the power of alpha as in Eq. (2):

2 $${p}_{{w}_{i}}={p}_{{o}_{i}}* {{f}_{i}}^{\alpha }$$

Where ${p}_{{w}_{i}}$ is the whitened power at the ith frequency, ${p}_{{o}_{i}}$ is the original power at the ith frequency bin, *f*_*i*_ is the ith frequency, and α is the aperiodic exponent calculated by applying FOOOF to the PSD data^20^. This whitening method was used for both LFP and spiking PSD (Supplementary Fig. 1f). The corresponding whitened PSDs are called “whitened LFP PSD” and “whitened SPK PSD”, respectively. This whitening method will be referred to as “whitening in the frequency domain (pwelch-FOOOF-whitening)”.

### Time-domain whitening procedure

Classical whitening is done in the frequency domain, as detailed in the previous section. We also whiten our data in the time domain (https://www.mathworks.com/matlabcentral/fileexchange/65345-spectral-whitening). We applied the time-domain whitening technique to both the LFP and SPK signals (Supplementary Fig. 2). We first multiplied the time domain signal by an n-point symmetric Hann window (where n is the length of the signal) to diminish spectral leakage. The Fourier transform of this multiplied signal was then obtained with a fast Fourier transform (fft, MATLAB R2020b). The magnitude and phase of each element were extracted from this signal by computing the absolute value and the angle, respectively. The magnitude values, in the frequency range from 3 to 70 Hz, were then whitened by multiplying each magnitude by its frequency to the power of alpha as in Eq. 3:

3 $${m}_{{w}_{i}}={m}_{{o}_{i}}* {{f}_{i}}^{\alpha }$$

Where ${m}_{{w}_{i}}$ is the whitened magnitude at the i^th^ frequency bin, ${m}_{{o}_{i}}$ is the original magnitude at the ith frequency, *f*_*i*_ is the ith frequency, and α is the aperiodic exponent calculated from FOOOF on the magnitude data^20^. The modified signal was then transformed back to the time domain using MATLAB’s inverse fast Fourier transform function (ifft, MATLAB R2020b) to obtain the whitened signal, henceforth referred to as the “whitened LFP signal” and the “whitened SPK signal” (Supplementary Fig. 2e1, e2). The whitened PSD was then estimated from the whitened signal in each recording depth using the Welch method (MATLAB R2020b, pwelch). Henceforth, we will refer to this method as whitening in the time domain (FFT-FOOOF-whitening-iFFT-pwelch).

### Coherence analysis

The coherence analysis between the LFP and rectified spiking signals of the same microelectrodes was estimated using the magnitude squared coherence function (MATLAB R2020b) with a Hamming window of 2 s (resulting in a frequency resolution of 0.5 Hz) and 50% overlap. We show the coherence function over a frequency range from 3 to 70 Hz (Supplementary Fig. 2d). We performed the same coherence analysis on the whitened LFP and SPK signals using the same parameters, except that we limited the frequency range from 3 to 70 Hz already at the stage of the time-domain whitening since the value of aperiodic exponent (alpha) was obtained at this range (Supplementary Fig. 2f).

### Delimitating STN subregions

The Hidden Markov Model (HMM) algorithm was used to automatically detect the STN borders and subregions based on the spiking signal and the accuracy of this method was confirmed in our previous publication^33^. We used these results to define the regions in both the SPK and LFP analyses (Supplementary Fig. 1e1, e2). The HMM algorithm enforces sharp transitions between regions. To maximize the reliability of our subregion definition, we chose to exclude the 0.5 mm nearest to the border of each region, thereby establishing “safe boundaries”. Thus, in the STN motor and non-motor subregions, we excluded 0.5 mm nearest to the detected borders of both entry and exit. For the pre-STN, we excluded the final 0.5 mm preceding the exit. Our previous study indicated the subregion transition detection reliability is up to 100% when limiting Hits to error <2 mm and not smaller than 86% when error <1 mm. Therefore, the reliability of our subregion definition was larger than 86%. We averaged the PSD within the safe boundaries in each subregion. These averaged PSD from 3 to 70 Hz were used for the FOOOF analysis (Supplementary Fig. 1f).

### Testing the effects of the values of aperiodic and periodic components on the goodness of fit of the FOOOF algorithm

We simulated (based on the function (y = αx + b)) 3–70 Hz spectra without Gaussian periodic elements using aperiodic exponent (*α*) ranging from −0.25 to 2.25 (Supplementary Fig. 3 left subplots). The aperiodic offset was set to equal α, in line with our finding of a positive linear correlation between the offset and the exponent (Supplementary Fig. 4). We also simulated spectra with Gaussian periodic elements using the same aperiodic parameters (Supplementary Fig. 3, right subplots). In this situation, three periodic Gaussian elements with mean, standard deviation, and amplitude values of 18 ± 5 Hz and 1.5 log_10_(power), 25 ± 8 Hz and 3 log_10_(power), and 35 ± 5 Hz and 2 log_10_(power), respectively were added. Finally, a random noise with Gaussian distribution was added to each spectrum to achieve mean absolute error (range from 0.005 to 0.145).

FOOOF analysis was applied to those simulated spectra to obtain their *R*^2^ and MAE values. *R*^2^ values were transformed using inverse hyperbolic tangent. We repeated the above process for each permutation and combination of offset, α, and noise 20 times. *R*^2^ and MAE values were averaged, and their corresponding standard deviations were calculated. The averaged and the standard deviations of the *R*^2^ values were back-transformed using hyperbolic tangent. Only the average values are shown in Supplementary Fig. 3.

### Simulation of LFP and SPK activity modulated by Brown noise and β oscillations

We used the dsp.ColoredNoise function (MATLAB 2021a) to generate a Brown noise signal with a length of 8192 samples (simulating a 2-second signal with a sampling rate of 4096 samples per second). We applied a high-pass 2nd order Butterworth filter with a 0.1 Hz cutoff to imitate our patient data, which are real-time high-pass filtered at this frequency. We removed the first and last 2048 samples, leaving only the 4096 middle samples to avoid filter edge effects. We simulated the β signal by creating a 1-s (4096 samples per second) sine wave at 20 Hz with an amplitude of 0.5*SD(x) (where x is the Brownian noise signal). We added the beta signal to the Brown noise signal to create a beta-modulated signal. We then rectified the beta-modulated signal (i.e., took the absolute value of the signal) and subtracted the mean of the rectified signals (Supplementary Fig. 6a–d, left).

For the LFP and the SPK activity simulations (Supplementary Fig. 6f–i, left subplots), we defined a high amplitude beta signal, with an amplitude of 1.2*SD(x) to create a threshold at which the spikes would ride on the beta peaks rather than being influenced by low-frequency activity due to the Brown noise. We added this high amplitude β signal to the Brown noise signal (x) to generate the high amplitude β modulated “membrane potential” signal.

We then defined the spike threshold as the 60^th^ percentile of the high amplitude beta signal. In the regions where the amplitude of the high amplitude β modulated signal exceeded the threshold, we added simulated spikes. The added spikes follow a Poisson distributed probability with a mean of 6 spikes per beta peak. The spike signal was defined as a vector of zeros of the same length as the original signal, with zeros replaced by ones at the time stamps where spikes were generated. The spikes were multiplied by 3*max (high amplitude beta signal) and added to the high amplitude beta-modulated brown noise signal. This signal was then band-pass filtered with a 6th-order Butterworth filter at 300–2000 Hz. Finally, the filtered signal was rectified by taking the absolute operator.

The simulation PSDs were obtained by generating 1000 samples of the time domain signals of each type described above, estimating the spectral density of each using a periodogram (MATLAB 2021a) with a Hamming window of 1 s and NFFT = 4096. We performed a log10 transform on the resultant frequency domain signals and frequencies and averaged the results across the 1000 samples. The PSD results are plotted in Supplementary Fig. 6a–i on the right.

### Alignment of PSDs and spectrograms to the beta center frequency

The averaged PSD within the STN motor domain of each trajectory was used to detect the highest peak beta between 13 and 33 Hz. The frequency of this peak was defined as beta center frequency (*β*CF). The frequency of each site of this trajectory was shifted, so the resulting *β*CF equals 0 Hz. This alignment enables us to assess the relative distribution of power around the center frequency. The alignment to the *β*CF was applied to LFP and SPK PSDs and spectrograms, as well as to their coherence functions and coherograms (Fig. 5, and Supplementary Figs. 8, 10). We used both averaged trajectory PSD and single site PSD to align to the center frequency. Using the average PSD of a trajectory enhances the estimate’s accuracy since the frequency of beta oscillations along a single trajectory DLOR is highly stable^1^.

### Calculating bandwidths of beta oscillations

We used the normalized PSD (NPSD) to find the highest beta peak and its location from 13 to 33 Hz. Half the highest peak prominence (half-highest-prom) was used as the reference height for width measurement. The half-band width was calculated by finding the distance between the half-highest-prom and the left and right flanks of the beta oscillation. The powers around the half-highest-prom and their corresponding frequencies were used for linear fitting to get the left and right edges of the half-band width. The distance between the left (or right) edge and the location of the highest beta peak was called half-band-half-side width (Supplementary Fig. 11). The bandwidths were calculated at three levels. At the single site level (*n* = 9147), we calculated the bandwidths of beta oscillation separately for each site. At the level of a single trajectory (*n* = 308), we averaged the NPSD within the STN motor domain of each trajectory and calculated the beta bandwidth from the averaged NPSD. At the population level (*n* = 1), we used the average of the NPSD within the STN motor domain of all trajectories to calculate the beta oscillation bandwidth. A similar procedure was done for the 1/4 height and 3/4 height bandwidths and their half-side bandwidths.

### Evaluating predictive factors for the burden and treatment efficacy of Parkinson’s disease

Linear Regression and Generalized Linear Models (GLMs) were fitted to estimate the predictive value of individual and combined predictor families on response (predicted) variables: UPDRS III score off dopamine replacement therapy before surgery (DRT Off), the UPFRS III difference and percentage change due to medication before surgery (DRT Diff and DRT Per), UPDRS III change due to DBS after surgery (DBS Diff and DBS Per), and the subjective evaluation of the neurologist of the DBS outcome about 1 year after surgery (DBS Eva). Many studies reported a lack of correlation between Parkinson’s tremor symptoms and the akinetic/rigid symptoms and the effect of therapy (worse for DRT, better for STN DBS). We, therefore, divided our patient cohort into akinetic/rigid and tremor-dominant patients. We did several prediction models for the akinetic/rigid, the tremor, and the whole groups (Fig. 6 and Supplementary Fig. 13). In the akinetic/rigid group: the number of observations for the UPDRS III pre- and post-op is 45 and 30 patients, respectively; the number of observations for the neurologist’s evaluation of the DBS effect is 60 patients. In the tremor group, there are 54 observations for DRT Off, 53 each for the DRT Diff and DRT Per, 32 each for the DBS Diff and DBS Per, and 66 observations for the DBS Eva. For the whole group, the observation counts are as follows: 100 for DRT Off, 99 for both DRT Diff and DRT Per, 62 for each of DBS Diff and DBS Per, and 129 for DBS Eva.

The individual predictors are levodopa daily equivalent dose (LD), age (Ag), disease duration (DD), gender (Gd), LFP beta center frequency (LCF), SPK beta center frequency (SCF), LFP beta peak power (LP), SPK beta peak power (SP), the difference between LCF and SCF (LSCF), LP and SP (LSP), LFP offset (LO), SPK offset (SO), LFP exponent (LE), SPK exponent (SE), as well as the difference between LO and SO (LSO), and LE and SE (LSE).

Predictor families were defined as Demography (DeF, consisting of LD, Ag, DD, and Gd), Aperiodic (ApF, consisting of LO, SO, LE, and SE), Periodic (PeF, consisting of LCF, SCF, LP, and SP), LFP (LFPF, consisted of LO, LE, LCF, and LP), SPK (SPKF, consisting of SO, SE, SCF, and SP), and the difference between LFP and SPK (LSF, consisting of LSCF, LSP, LSO, and LSE). All predictive and responsive variables were done with Z-normalization before the model fitting.

Modeling was done at different levels: individual predictors (Eq. (4), Fig. 6a); zeroth-order interactions of members in a family (Eq. (5), Fig. 6b and Supplementary Figs. 13–16); first-order interactions of each two members in a family (Eq. (6), Fig. 6c and Supplementary Figs. 13–16); higher-order interactions of each three or four members in a family (Eq. (7), Fig. 6d and Supplementary Figs. 13–16).

4 $$y={ax}+b$$

5 $$y={a}_{1}{x}_{1}+{a}_{2}{x}_{2}+{a}_{3}{x}_{3}+{a}_{4}{x}_{4}+b$$

6 $$y={a}_{1}{x}_{1}{x}_{2}+{a}_{2}{x}_{1}{x}_{3}+{a}_{3}{x}_{1}{x}_{4}+{a}_{4}{x}_{2}{x}_{3}+{a}_{5}{x}_{2}{x}_{4}+{a}_{6}{x}_{3}{x}_{4}+b$$

7 $$y={a}_{1}{x}_{1}{x}_{2}{x}_{3}+{a}_{2}{x}_{1}{x}_{2}{x}_{4}+{a}_{3}{x}_{1}{x}_{3}{x}_{4}+{a}_{4}{x}_{2}{x}_{3}{x}_{4}+{a}_{5}{x}_{1}{x}_{2}{x}_{3}{x}_{4}+b$$

Here, the *x* indicates individual predictors (LD, Ag, DD, Gd, LCF, SCF, LP, SP, LSCF, LSP, LO, SO, LE, SE, LSO, or LSE). The *y* indicates response variables (DRT Off, DRT Diff, DRT Per, DBS Diff, DBS Per, or DBS Eva). The *x*_1_*, x*_2_*, x*_3_, *and x*_4_ indicate the members in a predictor family.

The estimates of the goodness-of-fit (*R*^2^), the AIC, the statistical significance of the GLM fitting model (Model *P* value), the prediction quality (Coefficients), and the statistical significance of the coefficients (Coefficients’ *P* value) were calculated. All the values are shown in Fig. 6 using bar graphs for representative cases and in Supplementary Figs. 13–16 for all models, four normalization methods and the three groups of patients (akinetic-rigid, tremor dominant, and all). Both *R*^2^ and AIC are used to evaluate the goodness of GLM fitting. Higher *R*^2^ values suggested a stronger predictive capability. Lower AIC values indicated a more favorable balance between model fit and complexity. The statistical significance of the fitting model was represented by Model *P* value. The statistical significance of coefficients was represented by Coefficients’ *P* value, with lower values indicating better predictors. Statistical significance was set up at *p* < 0.05.

### Statistical analysis

Statistical analyses were performed using MATLAB (R2020b). If not specified, the data were presented as means ± standard deviation (SD) or means ± standard error of the mean (SEM), and statistical significance was set at *p* < 0.05. We used the Bonferroni correction to correct for multiple comparisons. We used the two-tailed Wilcoxon rank sum test to compare the PSD in each frequency point (Figs. 1, 3, 5, and Supplementary Figs. 5, 7, 8, 10). The two-tailed Wilcoxon signed-rank test was used for pairwise comparison of beta center frequency and half-band width (Figs. 4, 5, and Supplementary Figs. 4, 9–12). The N-way analysis of variance was used to analyze the difference in aperiodic parameters between LFP and SPK (Fig. 2, Supplementary Fig. 5, and Supplementary Tables 2–4).

### Large language model (LLM) declaration

We used ChatGPT 3.5 and 4.0 to enhance the English expression of our already-written manuscripts.

### Supplementary information

Supplementary material

## Data Availability

The data set for this publication is not publicly available due to privacy and ethical restrictions. The data is held by the Hadassah University Hospital, Jerusalem, Israel. Data sharing is restricted by the data sharing protocol of the Institutional Review Board (IRB) of the Hadassah Medical Organization, Jerusalem, Israel. The anonymized data is available to qualified academic investigators upon written request from the corresponding author (H.B.).

## Code availability

The code used in this study is available to qualified researchers upon reasonable request from the corresponding author (H.B.).
