# Supplementary material for: Subthalamic nucleus input-output dynamics are correlated with Parkinson’s burden and treatment efficacy
Source: NPJ Parkinsons Dis. 2024 Jun 15;10:117. doi: 10.1038/s41531-024-00737-8 (PMC11180194; doi:10.1038/s41531-024-00737-8)
Supplement: Supplementary file 1 — Supplementary material [file 41531_2024_737_MOESM1_ESM.pdf]

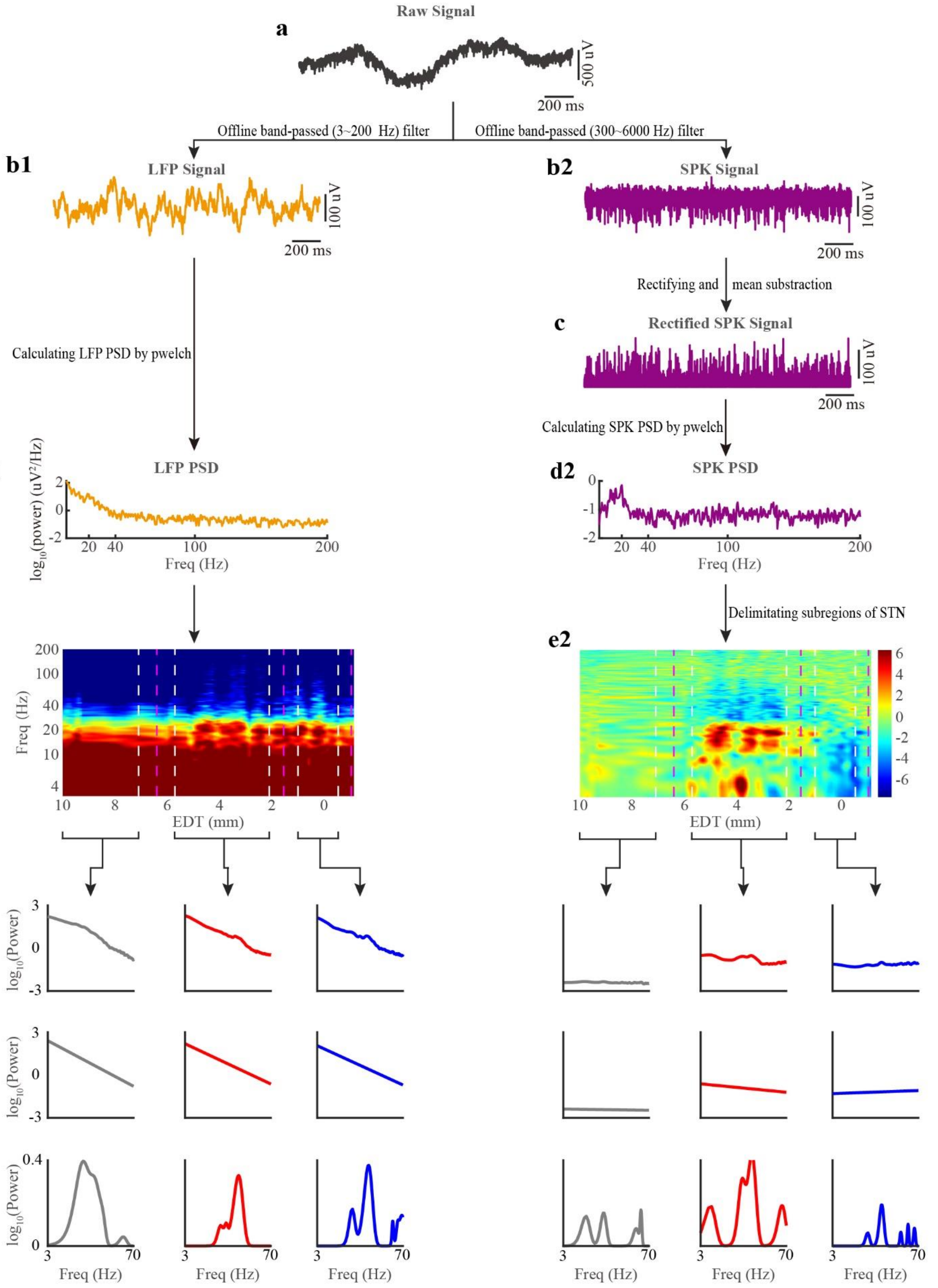

Supplementary Figure 1: **Electrophysiological pre-processing and analysis.** (**a**) Two seconds example of raw signal

band-passed filtered from 0.07 to 9000 Hz. (**b1**) The LFP signal is obtained by zero-phase band-pass filtering the raw signal from 3-200Hz. (**b2**) The spiking (SPK) signal is obtained by zero-phase band-pass filtering of the raw signal from 300 to 6000 Hz. (**c**) The rectified SPK signal is obtained by applying the absolute operator to the spiking signal and then subtracting the mean. (**d1**) The LFP power spectral density (PSD) in the range of 3 to 200 Hz is obtained by the pwelch function (MATLAB). (**d2**) The SPK PSD (3 to 200 Hz) is obtained by applying the pwelch function to the mean-subtracted rectified SPK signal. (**e1** and **e2**) Spectrograms and delimitating sub-regions of STN. The X-axis is the estimated distance to the target (EDT). The Y-axis is the frequency from 3 to 200 Hz (logarithmic scale). The spectrogram color scale represents $10*\log_{10}$(spectral power / average spectral power). The first and third vertical magenta dashed lines indicate the entry and exit of STN, respectively, and the second one represents the boundary between the dorsolateral oscillatory region (DLOR) and the ventromedial non-oscillatory region (VMNR). The vertical white dashed lines represent the safe 0.5 mm margins of each sub-region. STN borders were found by hidden Markov analysis (HMM) of neural spiking in **e2** and were copied to the same trajectory LFP data in **e1**. (**f**) The first row shows the averaged PSD (from 3 to 70 Hz) of each sub-region of a single trajectory. The aperiodic (row 2) and periodic (row 3) components of LFP and SPK activity of this single trajectory are obtained by applying FOOOF analysis to the averaged PSD in the first row. The columns indicate the regions: pre-STN (grey), STNDLOR (red), and STN VMNR (blue). See also Supplementary Figure 2.

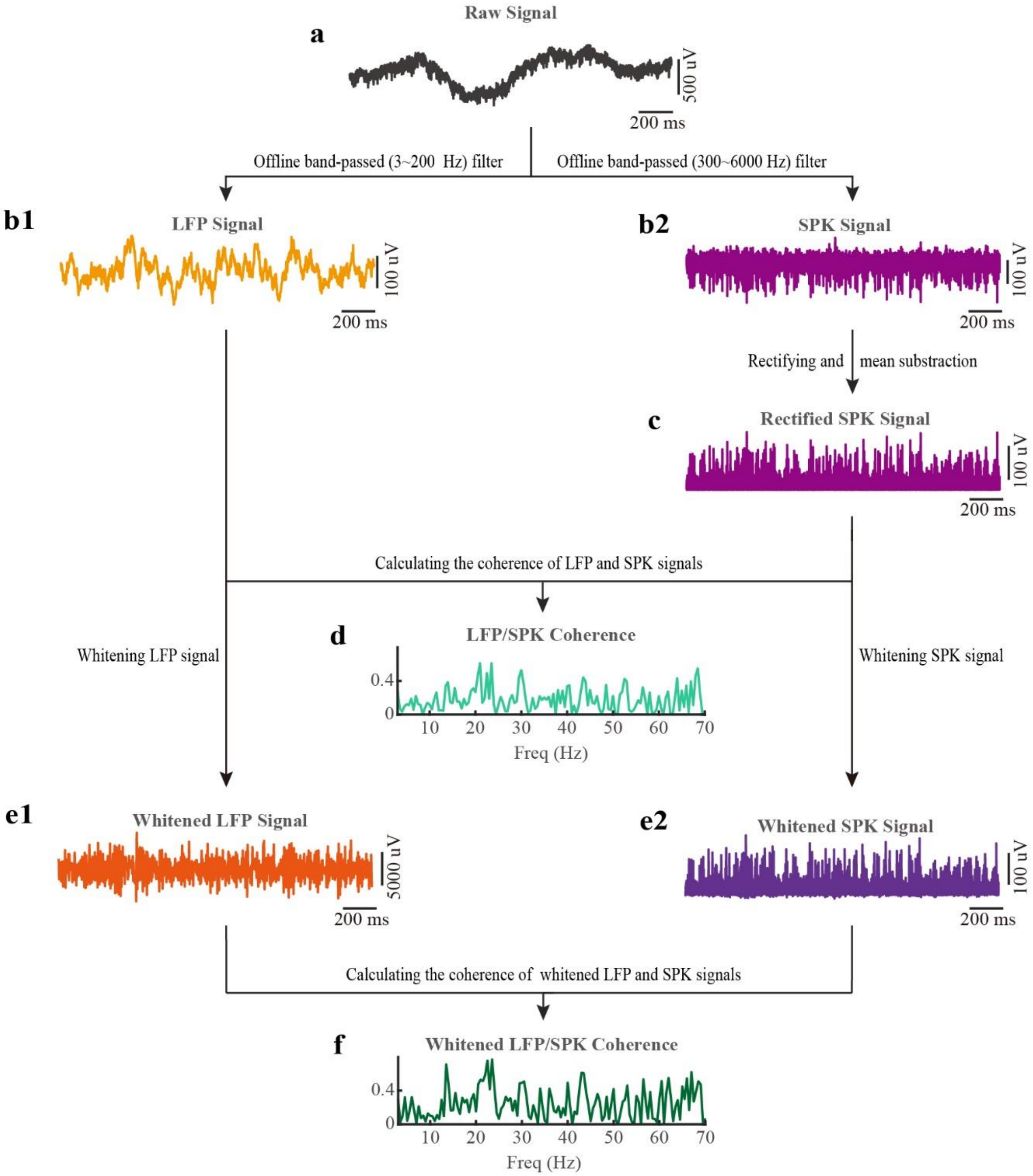

Supplementary Figure 2: **Calculating the coherence before and after whitening in the time domain.** (**a**) Two seconds example of raw signal band-pass filtered from 0.07 to 9000 Hz. (**b1**) The signal of LFP from 3 to 200 Hz was obtained by zero-phase filtering of the raw signal. (**b2**) The signal of spiking activity (discharge rate) was obtained by zero-phase filtering of the raw signal from 300 to 6000 Hz. (**c**) Applying the absolute operator to the spiking signal and then subtracting the mean created the rectified spiking signal. (**d**) The coherence of the simultaneously recorded LFP and rectified spiking signals of the same micro-electrode was estimated. (**e1** and **e2**) The whitening technique (equation (3), FFT-FOOOF-whitening-iFFT-pwelch procedure) was applied to the LFP and rectified spiking signals to obtain the whitened LFP signal and the whitened SPK signal. Note the different Y-scale of **e1**. The whitening process of the LFP ($\alpha = 2.2 \pm 0.40$) increased the power in the high-frequency band and amplitude of the LFP signal. (**f**) The whitened coherence of the simultaneously recorded whitened LFP and SPK signals of the same micro-electrode was calculated. Related to Supplementary Figure 1.

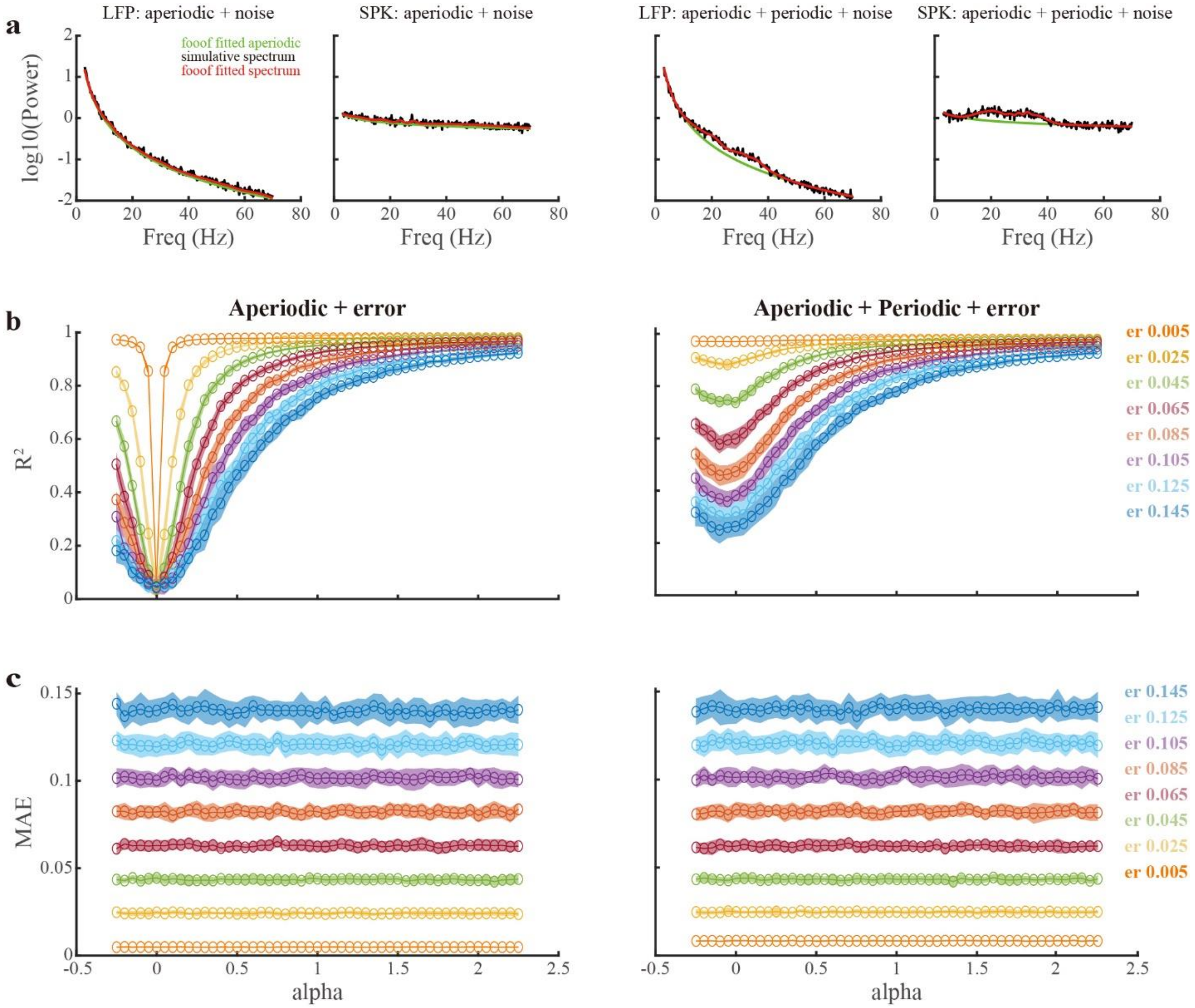

Supplementary Figure 3: **The absolute value of the aperiodic exponent (α) and periodic oscillations affect fitting R^2 values but not mean absolute error (MAE).** (**a**) Examples of simulated aperiodic LFP and SPK spectra (left) and aperiodic plus periodic components spectra (right). (**b**) In simulated spectra, as the absolute value of α decreases, the value of R^2 also decreases. The addition of Gaussian periodic elements increases R^2 values (right), and the addition of Gaussian distributed noise of increasing magnitudes (er, color-coded) lowers R^2 values. (**c**) α and Gaussian periodic elements don't change the MAE. As expected, the addition of simulated Gaussian distributed noise of increasing magnitudes increases the MAE. Related to Figures 1 and 2.

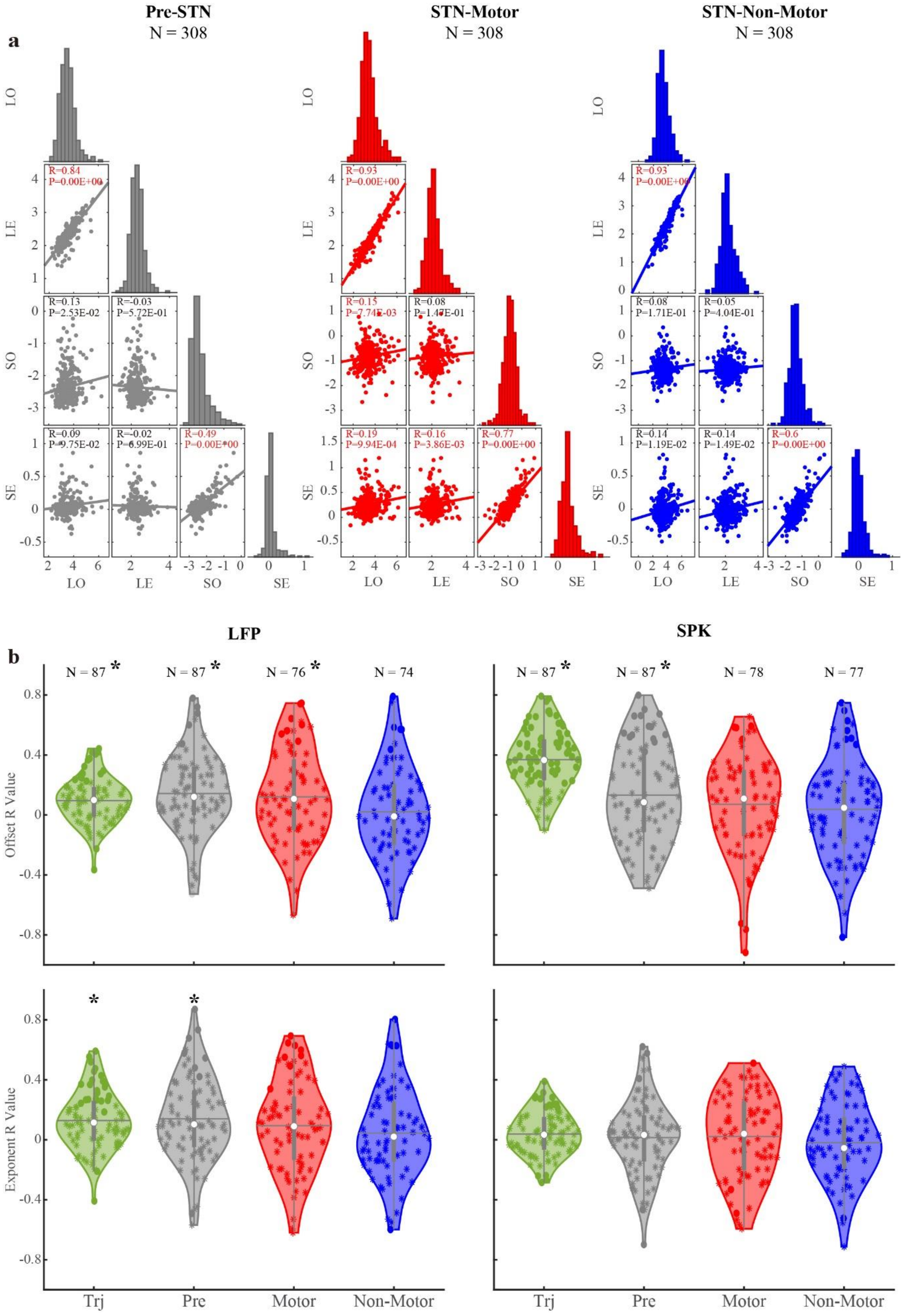

a
Pre-STN
N = 308
STN-Motor
N = 308
STN-Non-Motor
N = 308
LO
LE
SO
SE
R=0.84
P=0.00E+00
R=0.13
P=2.53E-02
R=-0.03
P=5.72E-01
R=0.09
P=9.75E-02
R=-0.02
P=6.99E-01
R=0.49
P=0.00E+00
R=0.93
P=0.00E+00
R=0.15
P=7.74E-03
R=0.08
P=1.47E-01
R=0.19
P=9.94E-04
R=0.16
P=3.86E-03
R=0.77
P=0.00E+00
R=0.93
P=0.00E+00
R=0.08
P=1.71E-01
R=0.05
P=4.04E-01
R=0.14
P=1.19E-02
R=0.14
P=1.49E-02
R=0.6
P=0.00E+00
b
LFP
SPK
N = 87 *
N = 87 *
N = 76 *
N = 74
N = 87 *
N = 87 *
N = 78
N = 77
Offset R Value
Exponent R Value
Trj
Pre
Motor
Non-Motor
Trj
Pre
Motor
Non-Motor

Supplementary Figure 4: **The correlation of LFP and SPK aperiodic parameters.** (**a**) The correlation between offset and the exponent aperiodic parameters of LFP and SPK in the three subthalamic subregions. LO: LFP offset; LE: LFP exponent; SO: SPK offset; SE: SPK exponent. R indicates the correlation coefficient. P indicates the statistical probability of the regression line having a slope different from zero. Pearson's correlation coefficient was used, and the Bonferroni correction was used for significance testing (when $p < 0.05/6 = 0.0083$, R and P values are typed in red). The distribution histograms of the single parameters are plotted along the diagonal of each subplot. (**b**) The correlation of the same aperiodic parameters (offset-offset and exponent-exponent) between pairs of simultaneously recorded sites. Trj, Pre, Motor and Non-Motor indicate the sites are from the whole trajectory (three subthalamic subregions, green), Pre-STN (grey), motor subdomain of STN (red), and the non-motor subdomain of STN (blue), respectively. The black star (*) above the violin indicates a significant difference between the simultaneous and correlation coefficient after 100 shuffling iterations. Related to Figure 2.

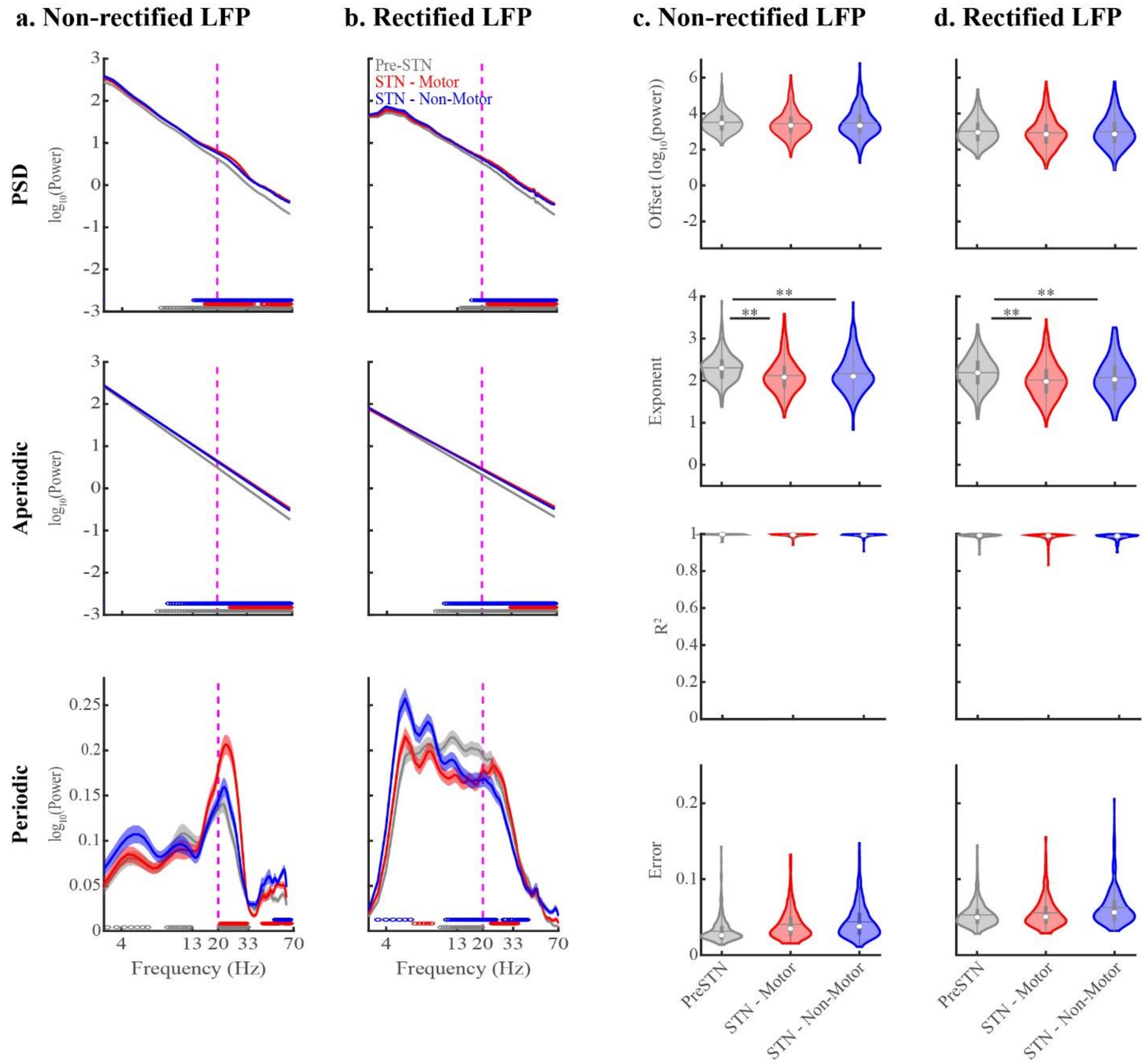

Supplementary Figure 5: **The aperiodic parameters of non-rectified and rectified LFP are qualitatively similar.** (**a**) Non-rectified LFP PSD and its aperiodic and periodic components in the pre-STN, STN motor, and non-motor sub-regions (grey, red, and blue lines, respectively). (**b**) Rectified LFP PSD and its aperiodic and periodic components in the STN sub-regions. Their SEMs are indicated by shade lines with corresponding colors. The circles above the x-axes represent significant differences between the pre-STN and STN motor subdomain (grey), the STN motor and non-motor subdomain (red), and the STN non-motor and pre-STN (blue) in corresponding frequency points. We used the Wilcoxon rank sum test and applied the Bonferroni correction. (**c**) Aperiodic parameters (offset and exponent) and assessment of the goodness of fit (R^2 and error) of the FOOOF analysis of non-rectified LFP in the three sub-regions. (**d**) Aperiodic parameters and goodness of fit assessment of the FOOOF analysis of rectified LFP in the three STN sub-regions. Color schemes are the same as in (**a**, **b**). The N-way analysis of variance (N-ANOVA) was used to analyze the difference between the aperiodic parameters. The two black asterisks indicate $p < 0.01$. The detailed multi-comparison of aperiodic parameters (offset and exponent) between non-rectified LFP and rectified LFP is shown in Supplementary Tables 3 and 4. Related to Figures 1 and 2.

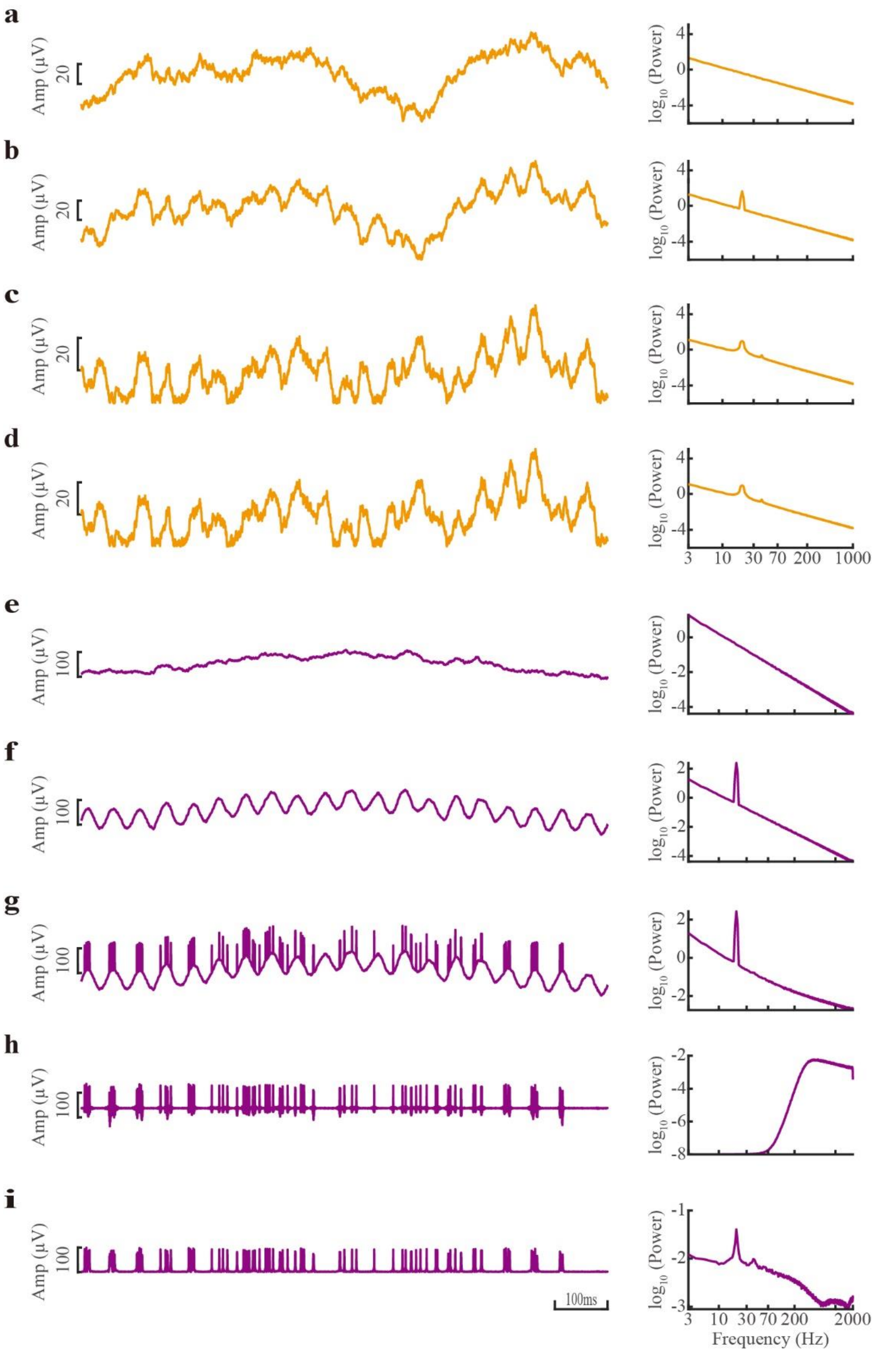

Supplementary Figure 6: **Simulation of brown noise with beta amplitude modulation of LFP and spiking signals in time (left) and frequency (right) domains.** (**a**) Brown noise. Left – example of a one-second trace of the analog time domain signal. Right – power spectral density of 1 second simulation. (**b**) Brown noise with beta (20 Hz) amplitude modulation. (**c**) The same as **b**, but rectified (taking the absolute value of the signal). (**d**) The same as **c**, but

subtracting its mean value. (**e**) Brown noise (note the lower resolution of the time-domain Y axis and frequency domain X (Freq) axis compared to **a**-**d**). (**f**) Brown noise and beta (20 Hz) amplitude modulation. (**g**) Beta-modulated Brown noise and Poisson spiking following a threshold crossing. (**h**) The same as **g**, but filtered at 300-2000Hz with a zero-phase 6th order Butterworth filter. (**i**) the same as **h**, but rectified (i.e., taking the absolute value of the signal and mean subtracted). Related to Figures 1 and 2.

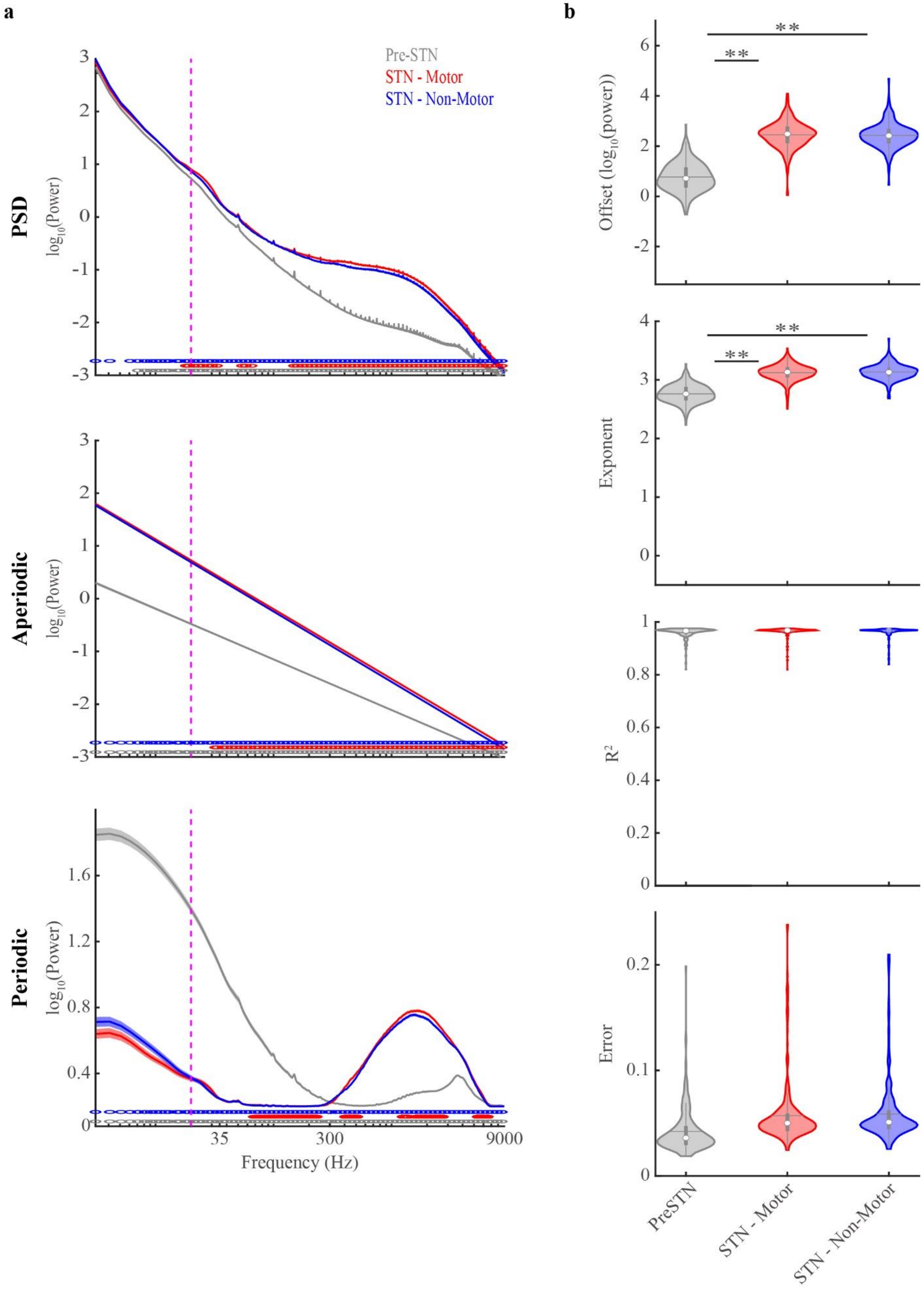
a
b
Pre-STN
STN - Motor
STN - Non-Motor
PSD
Aperiodic
Periodic
log10(Power)
Frequency (Hz)
35
300
9000
Offset (log10(power))
Exponent
R2
Error
**
PreSTN
STN - Motor
STN - Non-Motor

Supplementary Figure 7: **FOOOF analysis of broad-band (3-9000Hz) raw signal masks low frequency (3-40Hz) periodic oscillations of LFP and spiking activity.** (**a**) Population average PSD and aperiodic and periodic components (from top to bottom) of raw broadband signal in three STN sub-regions (Pre-STN, STN motor, and non-motor subdomains, in grey, red, and blue, respectively). Their SEMs are indicated by shade lines with corresponding colors. The circles above the X-axes represent the significant difference between the pre-STN and STN (grey), between the DLOR and VMNR (red), and between the VMNR and pre-STN (blue) in corresponding frequencies. (**b**) Offset, exponent, $R^{2,}$ and error of broad-band raw signal in the three STN sub-regions. The grey/red/blue violins indicate pre-STN, STN-DLOR and STN-VMNR, respectively. The two black asterisks from top to bottom indicate p = $4.28*10^{-96}$, $2.89*10^{-94}$, $4.35*10^{-90}$, and $1.18*10^{-86}$ (calculated using the Wilcoxon rank sum test and Bonferroni correction). Related to Figures 1 and 2.

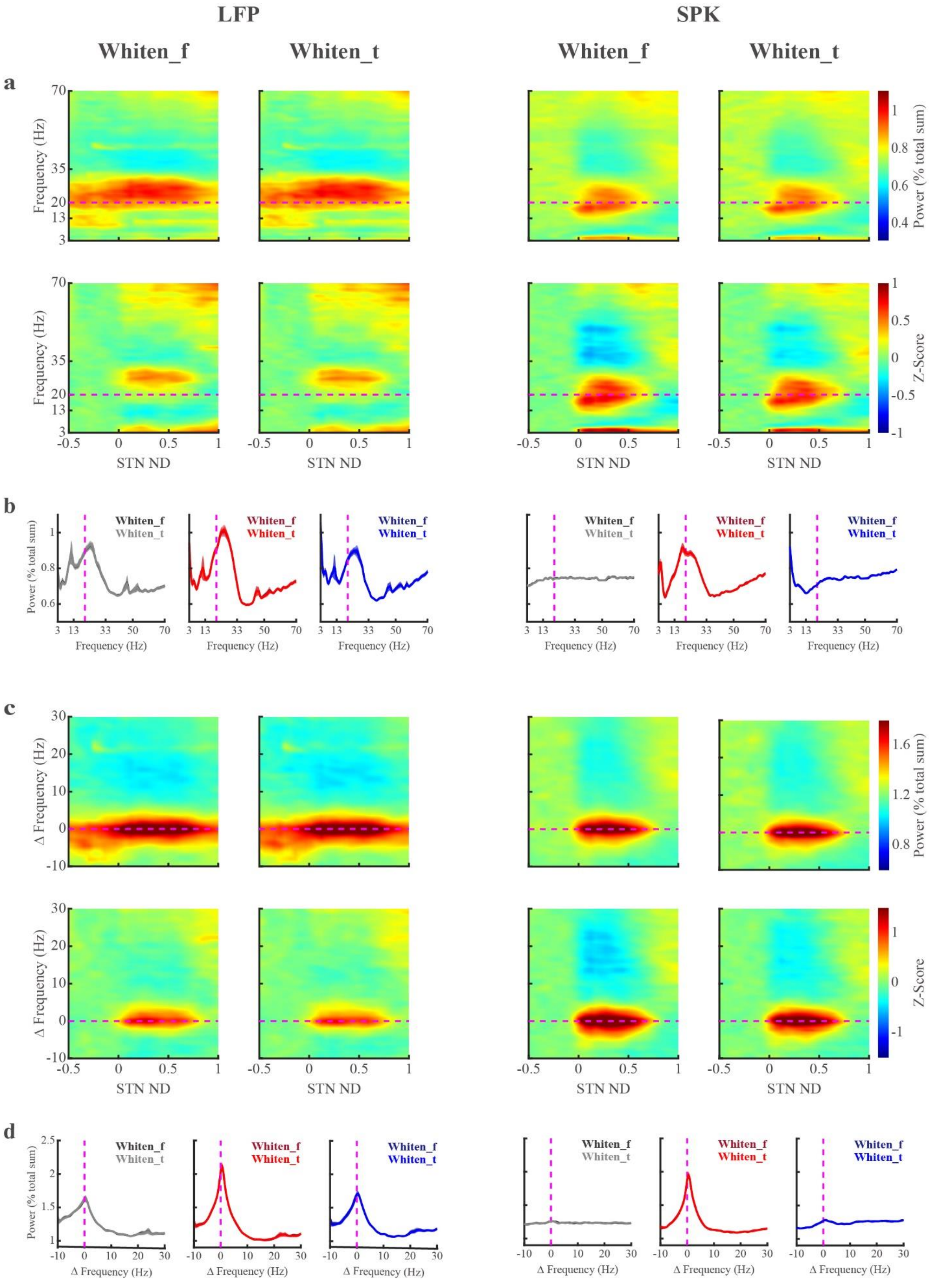

LFP
SPK
Whiten_f
Whiten_t
Whiten_f
Whiten_t
a
b
c
d
Frequency (Hz)
Power (% total sum)
Z-Score
STN ND
Frequency (Hz)
Δ Frequency (Hz)

Supplementary Figure 8: **There is no significant difference in average spectrograms and power spectrum densities obtained with the frequency-domain and time-domain whitening methods.** The spectrograms and power spectrum densities of LFP and SPK are on the left and right panels, respectively. (**a** and **c**) Spectrograms in columns 1 and 3 are whitened in the frequency domain, and in columns 2 and 4 are whitened in the time domain. The frequencies (y-axis) in **c** are aligned to the peak beta frequency. The horizontal magenta dashed lines in **a** and **c** are the reference lines of 20 Hz and the aligned peak beta frequency (ΔFrequency = 0 Hz), respectively. X-axis is the STN normalized distance (STN ND, same as in Figure 3). In the upper row of **a** and **c**, the spectrograms are normalized by frequency, and the color scale indicates the percentage of total power. In the lower row of **a** and **c**, the spectrograms are normalized by frequency and distance, and the color scale represents the standard deviation from the mean value of the first 10 depths in pre-STN (z-score). (**b** and **d**) The LFP power spectrum densities normalized by frequency (i.e., total power in the tested frequency range) are compared between the two whitening methods in three STN sub-regions (the left panel in **b** and **d**). The SPK power spectrum densities normalized by frequency are compared between the two whitening methods in three STN sub-regions (the right panel in **b** and **d**). The averaged power spectrum densities in **b** and **d** are before and after alignment to the peak beta frequency, respectively. The grey/red/blue lines indicate the average power spectrums in pre-STN/STN motor /STN non-motor sub-regions whitened in the frequency domain (dark) and in the time domain (light), respectively. Their SEMs are indicated by shade lines with corresponding colors. The two whitening methods have no significant differences in corresponding frequency points (Wilcoxon rank sum test). Whiten_f: whiten in the frequency domain. Whiten_t: whiten in the time domain. Related to Figure 3.

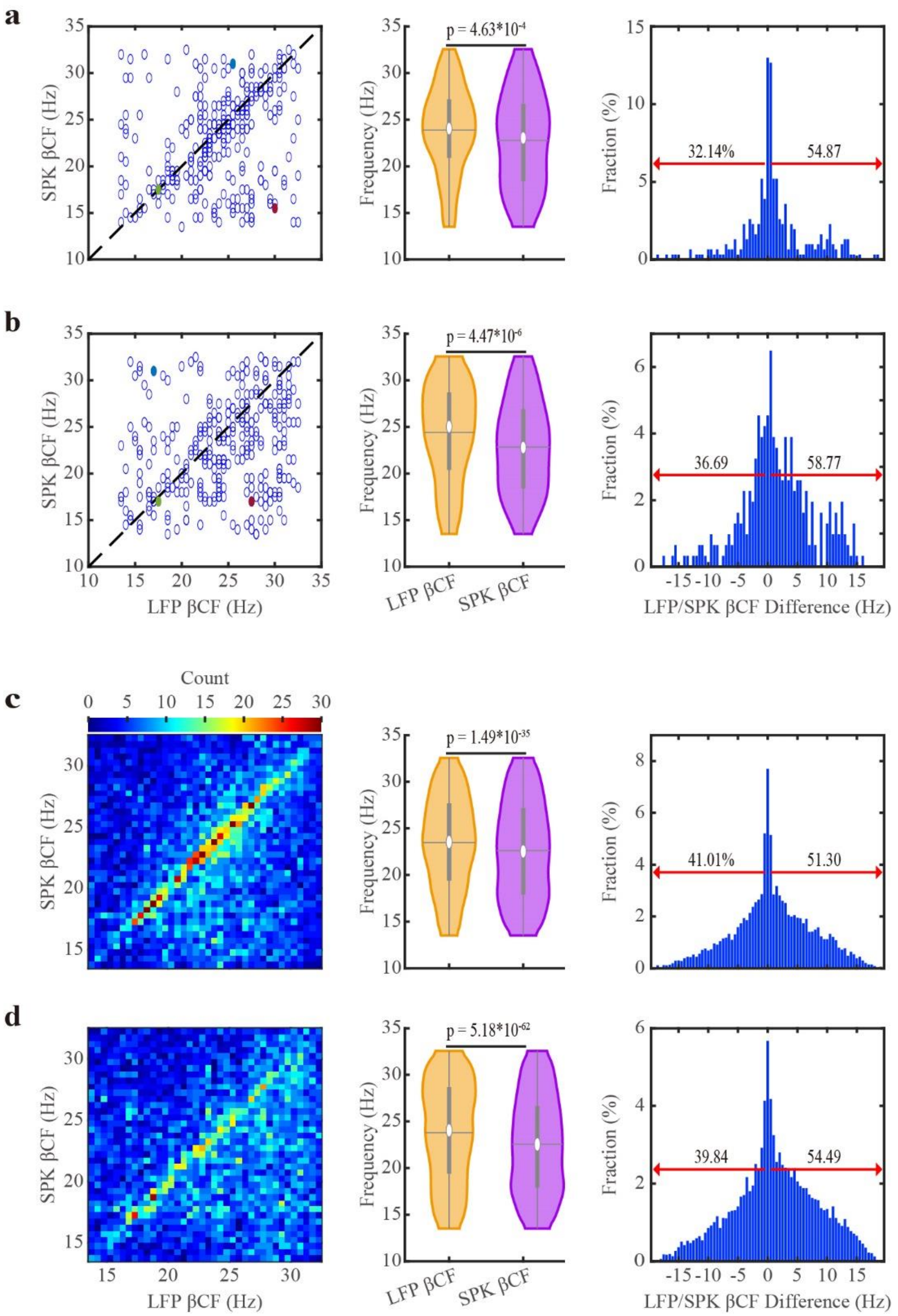

Supplementary Figure 9: **The downshift of the center frequency of beta oscillations in the time domain whitening method is similar to the downshift detected in the frequency domain whitening method.** (**a** and **b**) The unit to get beta center frequencies (βCFs) is trajectory. (**c** and **d**) The unit to get βCFs is single recording site. (**a** and **c**) The βCFs are obtained from the frequency-normalized power spectra. (**b** and **d**) The βCFs are obtained from the frequency- and distance-normalized power spectra. The dark dashed lines in the left column of **a** and **b** are the diagonal lines where $x = y$. In the middle panel, the violins demonstrate the distribution of βCFs of LFP and SPK. The significance levels indicating the difference between the LFP and SPK βCF distributions (shown in the violin plots) were calculated by the Wilcoxon signed rank test. In the right column, the red arrows indicate the percentage of SPK βCFs that were upshifted (left) and downshifted (right) compared to the corresponding LFP βCFs. The filled circles in red, green, and

blue in the left column of **a** and **b** are the same examples marked in Figure 4. Related to Figure 4.

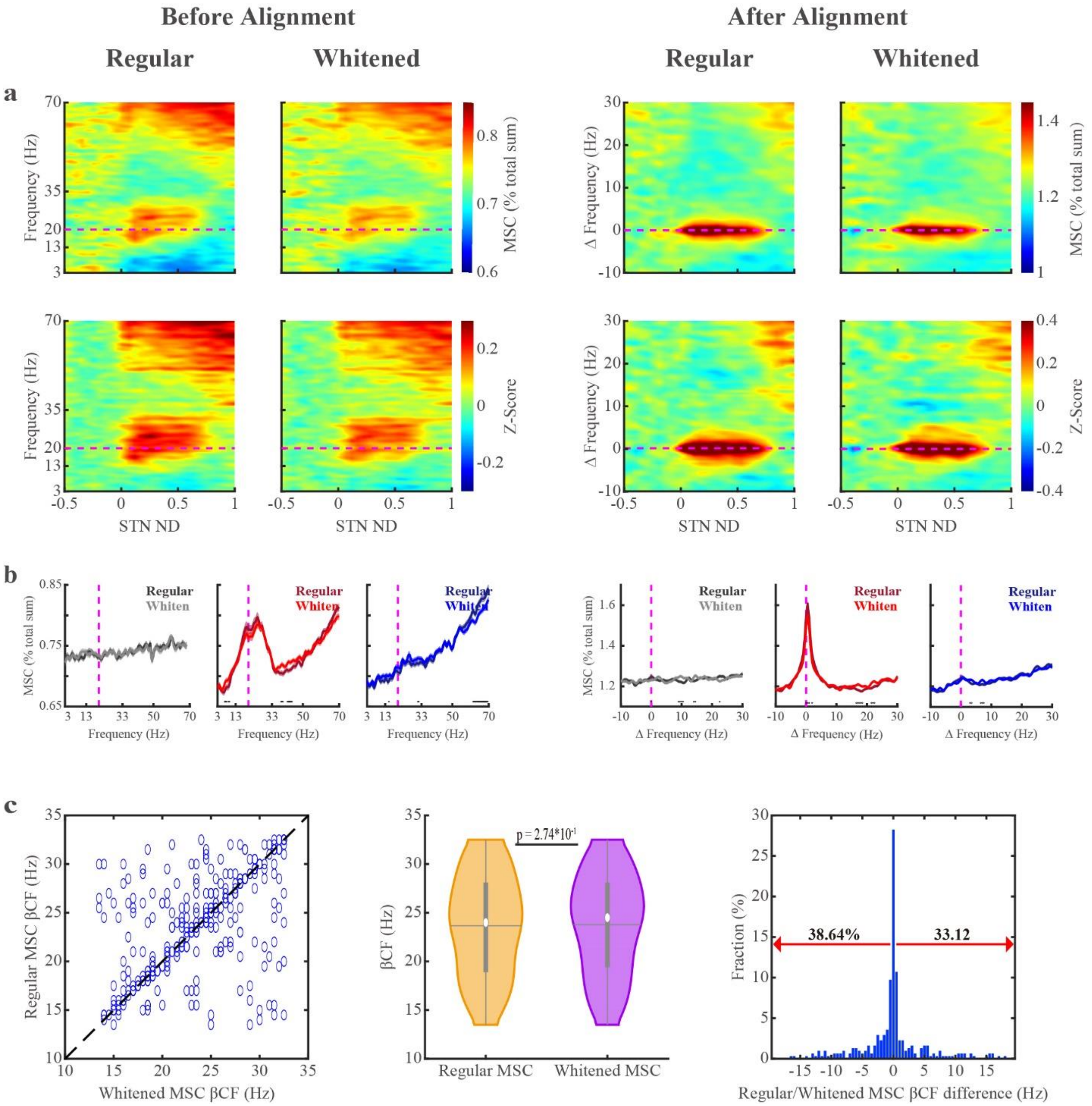

Supplementary Figure 10: **The regular and whitened coherograms reveal a narrow overlap of the distribution of LFP and SPK beta oscillations in the motor subregion of the subthalamic nucleus.** (**a**) The coherograms without and with frequency alignment are on the left and right panels of **a**, respectively. The regular and whitened coherograms before frequency alignment to the peak beta frequency (left panel) are normalized by frequency (top) and by frequency and distance (bottom). The coherograms aligned to the peak beta frequency (right panel) are normalized by frequency and by frequency and distance (first and second rows), respectively. The horizontal magenta dashed lines on the left and right panels of **a** are the reference line of 20 Hz and peak beta frequency (ΔFrequency = 0 Hz), respectively. The color scale in the first row of **a** indicates the percentage of total magnitude-square coherence (MSC). The color scale in the second row of **a** represents the standard deviation from the mean value of the first 10 depths in pre-STN (z-score). (**b**) The comparison of regular and whitened coherence functions in the three STN sub-regions. The dark and light lines indicate the averaged regular and whitened coherence in the pre-STN (grey), STN motor domain (red), and STN non-motor domain (blue), respectively. Their SEMs are indicated by shade lines with corresponding colors. The black circles above the X-axis represent significant differences (Wilcoxon rank sum test) between regular and

whitened coherence in corresponding frequencies. (**c**) The beta center frequencies (βCFs) are obtained from regular and whitened coherence normalized by frequency. The dark dashed line in the left sub-plot is the diagonal line. The Wilcoxon signed-rank test was used to calculate the statistical differences between the βCFs of regular and whitened coherence (middle subplot). The red arrows in the right subplot indicate the percentage of βCFs of regular coherence that are smaller (left) or greater (right) than that of the whitened coherence. Related to Figure 4.

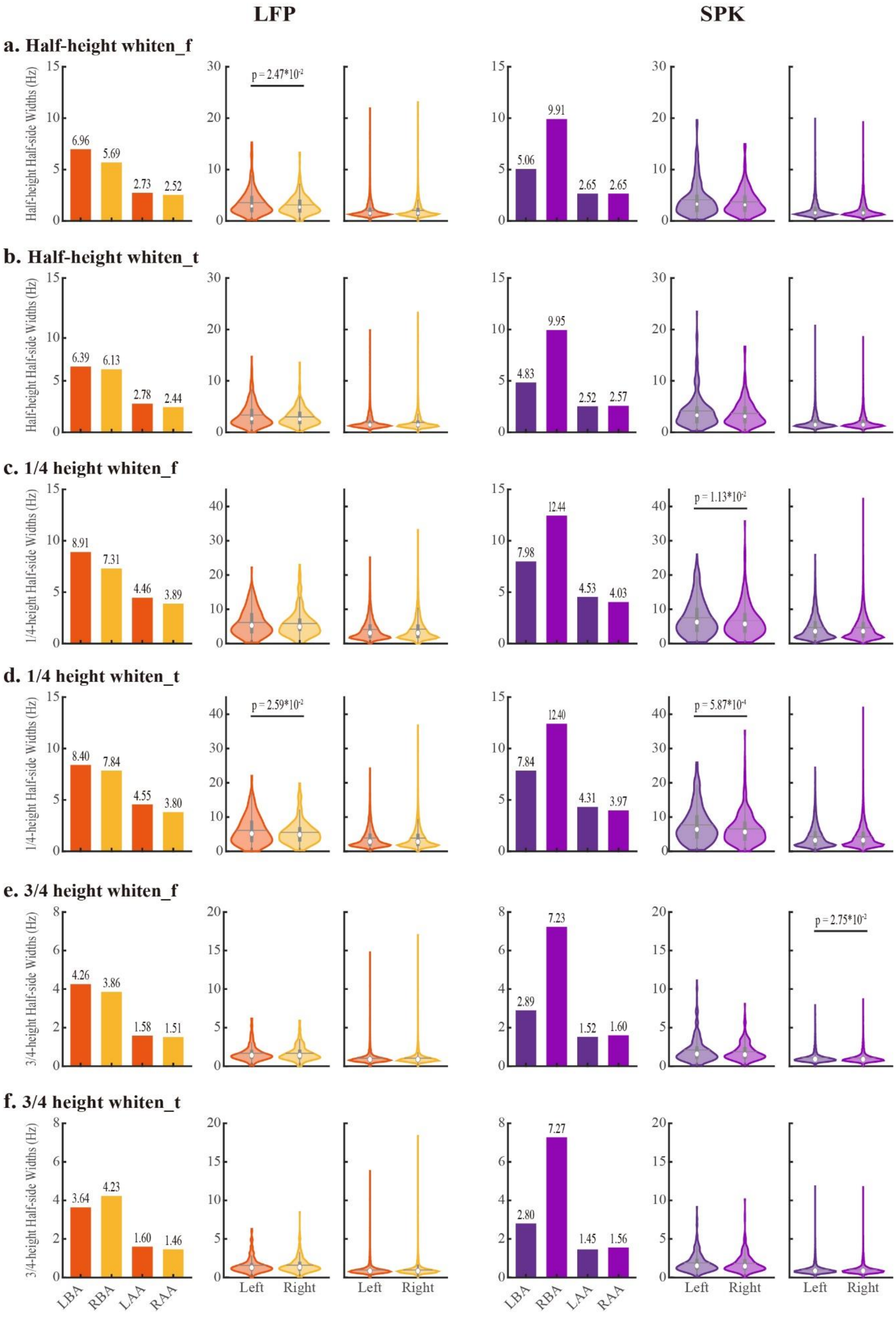
LFP
SPK
a. Half-height whiten_f
Half-height Half-side Widths (Hz)
6.96
5.69
2.73
2.52
p = 2.47*10^-2
5.06
9.91
2.65
2.65
b. Half-height whiten_t
Half-height Half-side Widths (Hz)
6.39
6.13
2.78
2.44
4.83
9.95
2.52
2.57
c. 1/4 height whiten_f
1/4-height Half-side Widths (Hz)
8.91
7.31
4.46
3.89
7.98
12.44
4.53
4.03
p = 1.13*10^-2
d. 1/4 height whiten_t
1/4-height Half-side Widths (Hz)
8.40
7.84
4.55
3.80
p = 2.59*10^-2
7.84
12.40
4.31
3.97
p = 5.87*10^-4
e. 3/4 height whiten_f
3/4-height Half-side Widths (Hz)
4.26
3.86
1.58
1.51
2.89
7.23
1.52
1.60
p = 2.75*10^-2
f. 3/4 height whiten_t
3/4-height Half-side Widths (Hz)
3.64
4.23
1.60
1.46
2.80
7.27
1.45
1.56
LBA
RBA
LAA
RAA
Left
Right

Supplementary Figure 11: **Symmetrical distribution of left and right half-band widths of beta oscillations of LFP and spiking (SPK) activity in single recording sites but not in the population.** LBA: left before alignment; RBA: right before alignment; LAA: left after alignment; RAA: right after alignment. (**a**) The left and right half-band widths of LFP and SPK beta oscillations whitened in the frequency domain. (**b**) The left and right half-band widths of LFP and SPK beta oscillations whitened in the time domain. (**c**) The left and right 1/4 height half-side bandwidths of LFP and SPK beta oscillations whitened in the frequency domain. (**d**) The left and right 1/4 height half-side bandwidths of LFP and SPK beta oscillations whitened in the time domain. (**e**) The left and right 3/4 height half-side bandwidths of the LFP and SPK beta oscillations whitened in the frequency domain. (**f**) The left and right 3/4 height half-side bandwidths of the LFP and SPK beta oscillations whitened in the time domain. In the first column of each subplot, the first two bars represent the population values without frequency alignment, and the last two bars represent the population values with frequency alignment. In the second column, each data point represents the averaged power spectrum in DLOR for each trajectory. In the third column of each graph, each data point represents the power spectrum in a single recording site. The power spectrum used for calculating the beta bandwidths is normalized by frequency. The left (dark) and right (light) side bandwidths of LFP and SPK are shown in orange and purple, respectively. We used the Wilcoxon signed rank test to calculate the statistical significance of the differences between right and left half-band widths of beta oscillations. Related to Figure 5.

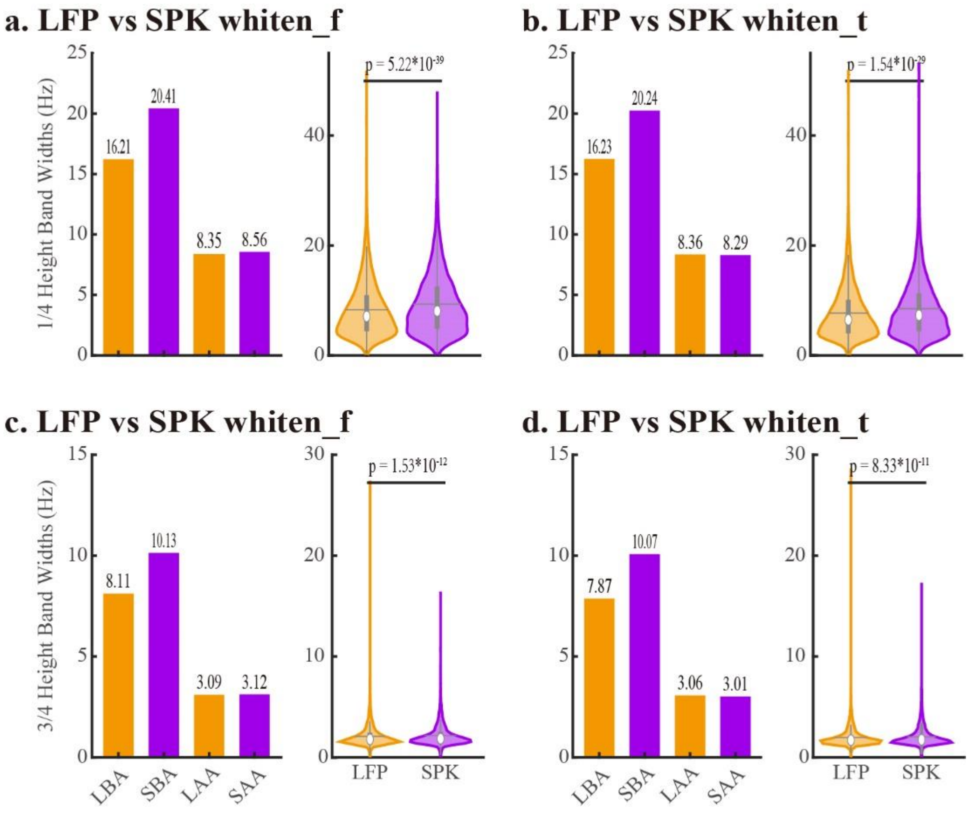

Supplementary Figure 12: **The comparison of both 1/4 and 3/4 height bandwidths of β oscillations between SPK and LFP is qualitatively similar to that of half-band width in STN DLOR.** LBA: LFP before alignment; SBA: SPK before alignment; LAA: LFP after alignment; SAA: SPK after alignment. (**a**) The two subplots from left to right demonstrate the comparison of 1/4 height bandwidths of β oscillations between LFP and SPK, which are whitened in the frequency domain in population and single site unit, respectively. (**b**) The two subplots (from left to right) show the comparison of 1/4 height bandwidths of β oscillations between LFP and SPK, which are whitened in the time domain in population and single site unit, respectively. (**c**) The two subplots (from left to right) reveal the comparison of 3/4 height bandwidths of β oscillations between LFP and SPK, which are whitened in the frequency domain in population and single site units, respectively. (**d**) The two subplots (from left to right) show the comparison of 3/4 height bandwidths of β oscillations between LFP and SPK, which are whitened in the time domain in population and single site unit, respectively. Related to Figure 5.

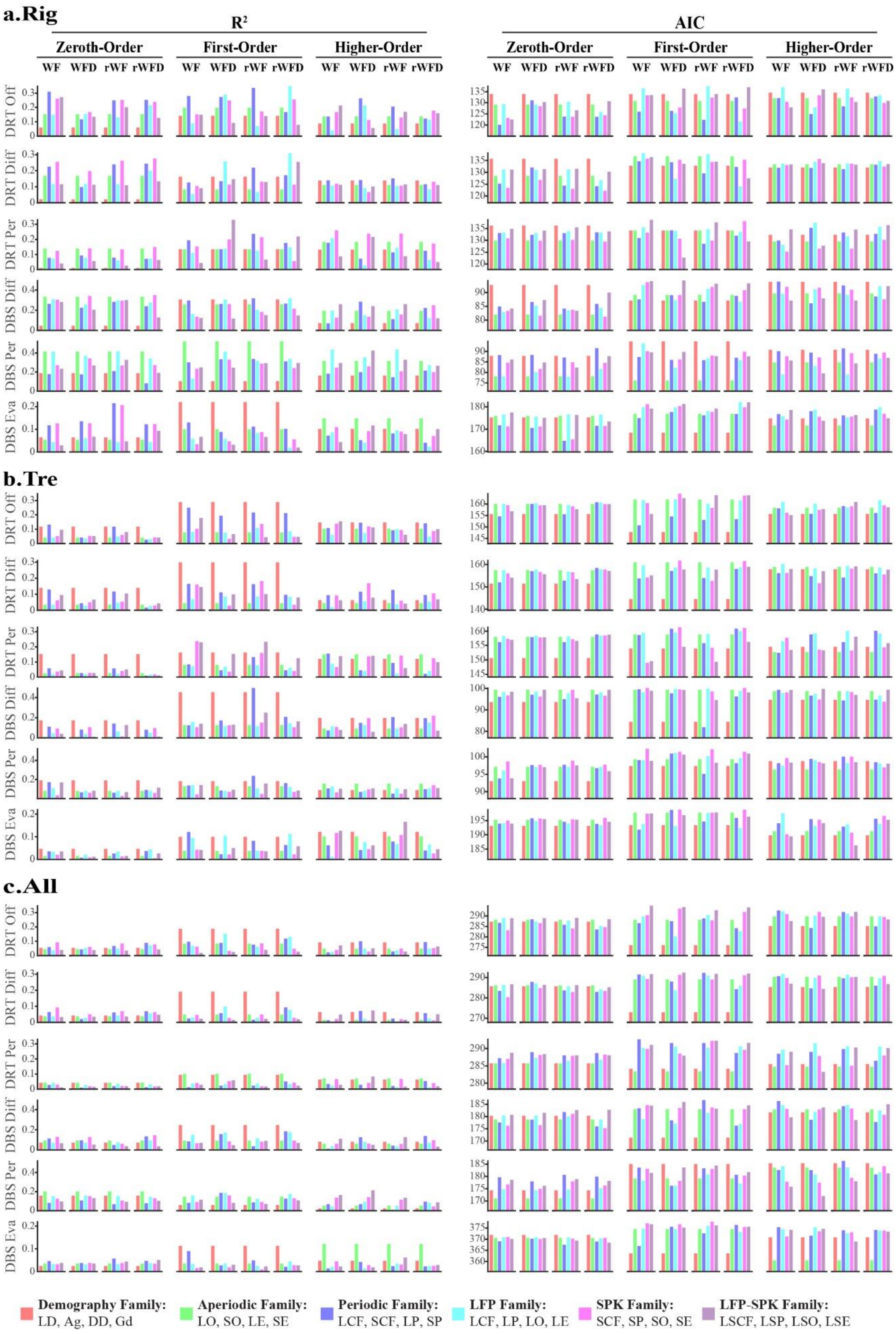

a.Rig
b.Tre
c.All
R^2
AIC
Zeroth-Order
First-Order
Higher-Order
WF
WFD
rWF
rWFD
DRT Off
DRT Diff
DRT Per
DBS Diff
DBS Per
DBS Eva
Demography Family: LD, Ag, DD, Gd
Aperiodic Family: LO, SO, LE, SE
Periodic Family: LCF, SCF, LP, SP
LFP Family: LCF, LP, LO, LE
SPK Family: SCF, SP, SO, SE
LFP-SPK Family: LSCF, LSP, LSO, LSE

Supplementary Figure 13: **R^2 and AIC of the comprehensive prediction analysis of the burden and treatment efficacy of Parkinson's disease.** We performed the prediction analysis on three groups of patients. (**a**) Patients who are primarily affected by akinesia and rigidity (Rig). (**b**) Patients with tremor symptoms (Tre). (**c**) All patients who underwent STN-DBS surgery. Six response metrics are included: DRT Off (UPDRS III, off dopamine replacement therapy), DRT Diff (change in UPDRS III score pre- and post-dopamine replacement therapy, DRT-off - DRT-on), DRT per (relative change in UPDRS III score pre- and post-dopamine replacement therapy, (DRT Off - DRT On)/DRT Off)), DBS Diff (change in UPDRS III score pre-dopamine replacement therapy and post-DBS therapy, Pre-DRT Off - Post-DRTonDBSon), DBS per (relative change in UPDRS III score pre-dopamine replacement therapy and post-DBS therapy, (Pre-DRT Off - Post-DRTonDBSon)/Pre-DRT Off)), DBS Eva (the general evaluations by neurologists of the efficacy therapy on a five-point scores from minus two to plus two). Predictor families are categorized as follows: Demography (red), Aperiodic (green), Periodic (blue), LFP (cyan), SPK (magenta), and the LFP-SPK differential (light purple). The elements of the predictor families are given at the bottom. Finally, signal processing conditions used are: WF (signals were whitened in the frequency domain and normalized by frequency), WFD (signals were whitened in the frequency domain and normalized by frequency and by distance), rWF (signals were whitened in the time domain and normalized by frequency), rWFD (signals were whitened in the time domain and normalized by frequency and by distance). The left and right panels demonstrate the Goodness-of-fit (R^2) and the Akaike Information Criterion (AIC) of general linear models (GLM) fitting, respectively. The fitting models include zeroth-order (equation (5)), first-order (equation (6)) and higher-order (equation (7)). Related to Figure 6.

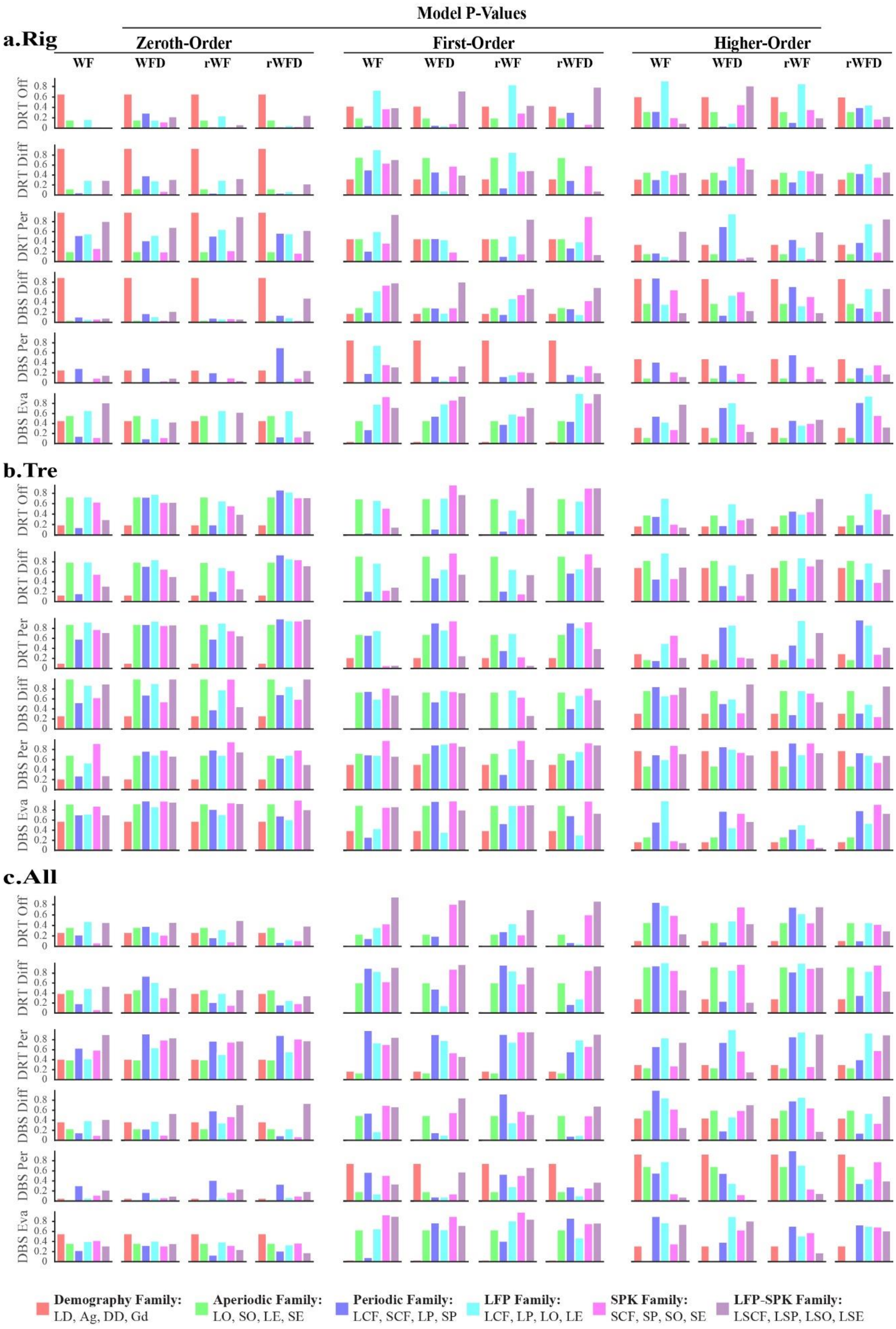

Model P-Values
a.Rig
Zeroth-Order
First-Order
Higher-Order
WF
WFD
rWF
rWFD
DRT Off
DRT Diff
DRT Per
DBS Diff
DBS Per
DBS Eva
b.Tre
c.All
Demography Family: LD, Ag, DD, Gd
Aperiodic Family: LO, SO, LE, SE
Periodic Family: LCF, SCF, LP, SP
LFP Family: LCF, LP, LO, LE
SPK Family: SCF, SP, SO, SE
LFP-SPK Family: LSCF, LSP, LSO, LSE

Supplementary Figure 14: **The Model P-Values of the comprehensive prediction analysis of the burden and treatment efficacy of Parkinson's disease.** (**a**) Patients who are primarily affected by akinesia and rigidity (Rig). (**b**) Patients with tremor symptoms (Tre). (**c**) All patients who underwent STN-DBS surgery. Six response metrics are included: DRT Off, DRT Diff, DRT per, DBS Diff, DBS per, and DBS Eva (they are the same as described in Supplementary Figure 13). Predictor families are categorized as follows: Demography (red), Aperiodic (green), Periodic (blue), LFP (cyan), SPK (magenta), and the LFP-SPK differential (light purple). The elements of the predictor families are given at the bottom. Signal processing conditions (WF, WFD, rWF and rWFD) are as described in Supplementary Figure 13. The general linear models (GLM) include zeroth-order (equation (5)), first-order (equation (6)) and higher-order (equation (7)). Related to Figure 6.

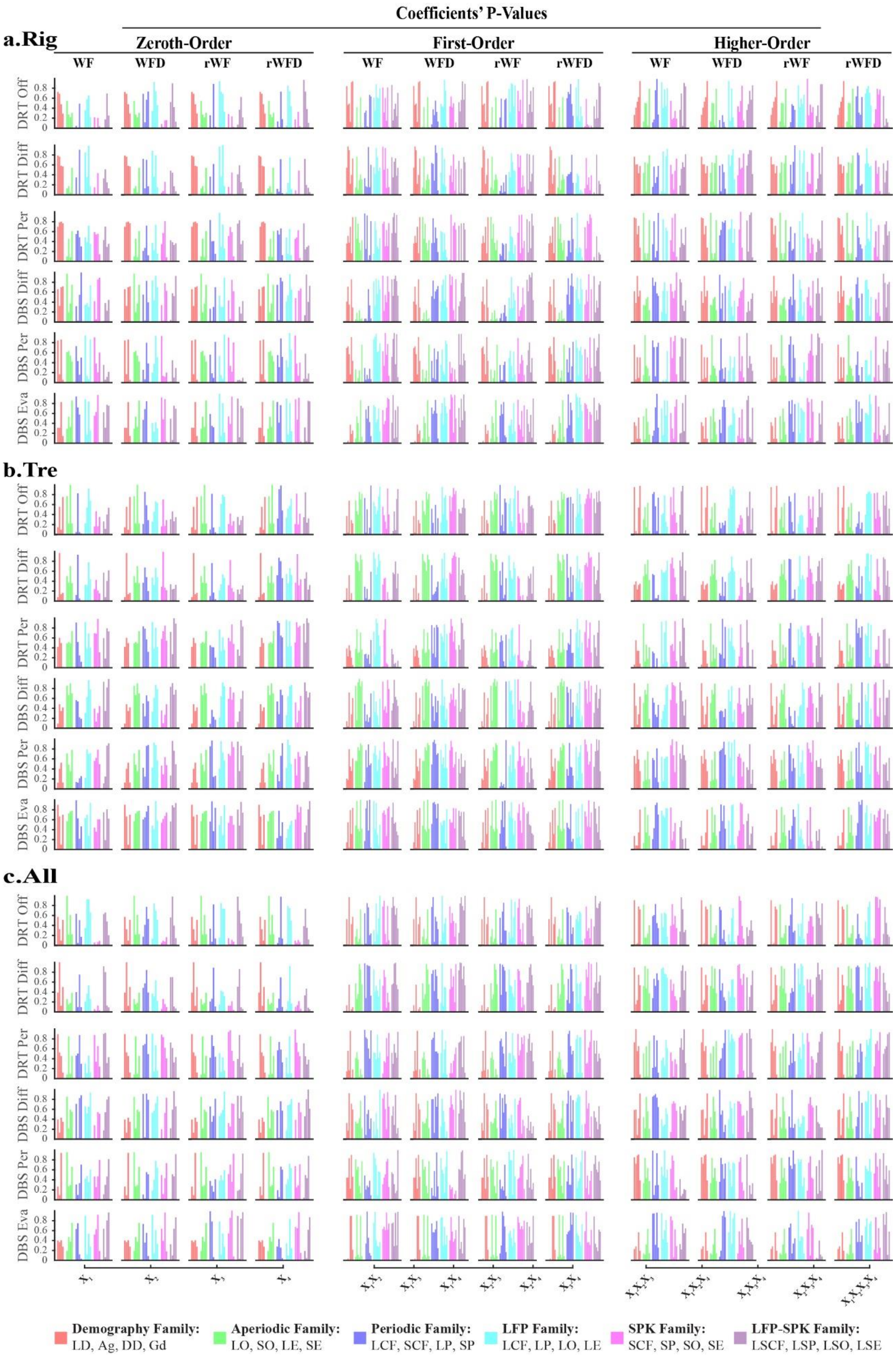

Coefficients' P-Values
a.Rig
b.Tre
c.All
Zeroth-Order
First-Order
Higher-Order
WF
WFD
rWF
rWFD
DRT Off
DRT Diff
DRT Per
DBS Diff
DBS Per
DBS Eva
Demography Family: LD, Ag, DD, Gd
Aperiodic Family: LO, SO, LE, SE
Periodic Family: LCF, SCF, LP, SP
LFP Family: LCF, LP, LO, LE
SPK Family: SCF, SP, SO, SE
LFP-SPK Family: LSCF, LSP, LSO, LSE

Supplementary Figure 15: **The Coefficients' P-Values of the comprehensive prediction analysis of the burden and treatment efficacy of Parkinson's disease.** (**a**) Patients who are primarily affected by akinesia and rigidity (Rig). (**b**) Patients with tremor symptoms (Tre). (**c**) All patients who underwent STN-DBS surgery. Six response metrics are included: DRT Off, DRT Diff, DRT per, DBS Diff, DBS per, and DBS Eva (they are the same as described in Supplementary Figure 13). Predictor families are categorized as follows: Demography (red), Aperiodic (green), Periodic (blue), LFP (cyan), SPK (magenta), and the LFP-SPK differential (light purple). The elements of the predictor families are given at the bottom. Signal processing conditions (WF, WFD, rWF and rWFD) are as described in Supplementary Figure 13. The general linear models (GLM) include zeroth-order (equation (5)), first-order (equation (6)) and higher-order (equation (7)). The $x_1,\ x_2,\ x_3\ and\ x_4$ indicate the members in a predictor family. The bars in a family indicate the coefficients' p-values of the corresponding elements marked above the color code. Related to Figure 6.

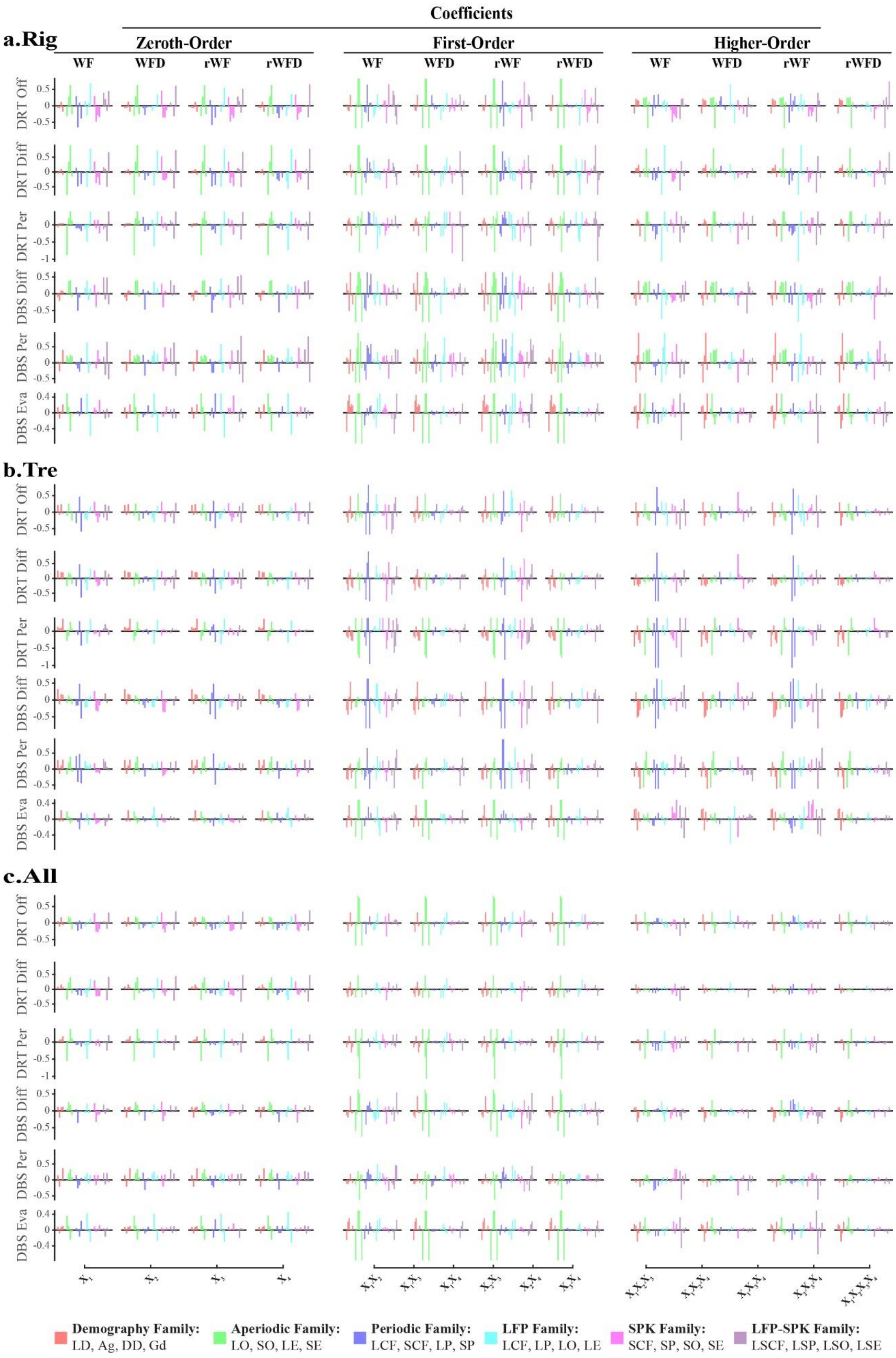
Coefficients
Zeroth-Order
First-Order
Higher-Order
WF
WFD
rWF
rWFD
a.Rig
b.Tre
c.All
DRT Off
DRT Diff
DRT Per
DBS Diff
DBS Per
DBS Eva
Demography Family:
LD, Ag, DD, Gd
Aperiodic Family:
LO, SO, LE, SE
Periodic Family:
LCF, SCF, LP, SP
LFP Family:
LCF, LP, LO, LE
SPK Family:
SCF, SP, SO, SE
LFP-SPK Family:
LSCF, LSP, LSO, LSE

Supplementary Figure 16: **The Coefficients of the comprehensive prediction analysis of the burden and treatment efficacy of Parkinson's disease.** (**a**) Patients who are primarily affected by akinesia and rigidity (Rig). (**b**) Patients with tremor symptoms (Tre). (**c**) All patients who underwent STN-DBS surgery. Six response metrics are included: DRT Off, DRT Diff, DRT per, DBS Diff, DBS per and DBS Eva (they are the same as described in Supplementary Figure 13). Predictor families are categorized as follows: Demography (red), Aperiodic (green), Periodic (blue), LFP (cyan), SPK (magenta), and the LFP-SPK differential (light purple). The elements of the predictor families are given at the bottom. Signal processing conditions (WF, WFD, rWF and rWFD) are as described in Supplementary Figure 13. The general linear models (GLM) include zeroth-order (equation (5)), first-order (equation (6)) and higher-order (equation (7)). The $x_1,\ x_2,\ x_3\ and\ x_4$ indicate the members in a predictor family. The bars in a family indicate the coefficients of the corresponding elements marked above the color bar. Related to Figure 6.

Supplementary Table 1. Demographics of patients, trajectories, and subthalamic recording sites

**Demographics**	**Results**	
Patients (N)	146	
Age (years) (Mean ± SD)	62.03 ± 9.63	
Gender (N, %)		
Male	100 (68.49%)	
Female	46 (31.51%)	
Disease Duration (years) (Mean ± SD)	10.17 ± 3.84	
Pre-operative LEDD (mg) (Mean ± SD)	1,020.71 ± 547.36 (n=145)	
Pre-operative UPDRS-III scores (Mean ± SD)		
Off medication	43.43 ± 12.73 (n=108)	
On medication	19.76 ± 8.99 (n=108)	
Post-operative scores (Mean ± SD)		
On stimulation/On medication UPDRS-III scores	17.65 ± 8.64 (n=72)	
On stimulation/On medication Neurologist's general evaluation	1.48 ± 0.72 (n=139)	
Total trajectories (N)	492	
Trajectories included (N)	308	
Right (E1, E2)	156 (68, 88)	
Left (E1, E2)	152 (72, 80)	
Recording Sites	LFP	SPK
Total sites (N)	42,680	42,680
Sites included (N, %)	25,822 (60.50%)	27,130 (63.57%)
Sites excluded (N, %)	16,858 (39.50%)	15,550 (36.43%)
Short signal length (N, %)	1,288 (3.02%)	1,288 (3.02%)
Outliers of RMS (N, %)	2,262 (5.30%)	434 (1.02%)
Trajectories excluded (N, %)	13,308 (31.18%)	13,828 (32.40%)
The length of sub-regions (mm) (Mean ± SD)	LFP	SPK
Pre-STN	4.35 ± 1.51	4.40 ± 1.50
STN motor domain (DLOR)	3.03 ± 1.08	3.06 ± 1.09
STN non-motor domain (VMNR)	2.63 ± 0.96	2.63 ± 0.96
DLOR-Per	0.53 ± 0.14	0.53 ± 0.14

The neurologist's general evaluation of the DBS outcome is based on subjective grading of the clinical status about a year after the procedure (+2: Excellent, +1: Very good, 0: Satisfactory, -1: Insufficient, -2: Poor). Pre- and Post-operative clinical scores were unavailable for all patients. In these cases, n depicts the number of available patients.

Abbreviations: STN: Subthalamic nucleus. SD: Standard deviation. N: number. LEDD: levodopa equivalent daily dose. E1: electrode 1 (Ben-Gun posterior location). E2: electrode 2 (Ben-Gun central location). DLOR: Dorsal lateral oscillatory region. VMNR: Ventral lateral non-oscillatory region. DLOR-Per: The percentage of DLOR length out of the total STN length (DLOR+VMNR)

**Supplementary Table 2: The multi-comparison of aperiodic parameters between LFP and SPK in the three subregions of STN**						
**Parameters**	**Group 1**	**Group 2**	**Lower Limit**	**Mean (Group 1) – mean (Group 2)**	**Upper Limit**	**P value**
**LFP vs SPK Offset**	LFP pre-STN	SPK pre-STN	5.74	5.88	6.01	**$2.07 * 10^{-08}$**
	LFP pre-STN	LFP DLOR	-0.06	0.07	0.21	0.65
	LFP pre-STN	SPK DLOR	4.20	4.34	4.48	**$2.07 * 10^{-08}$**
	LFP pre-STN	LFP VMNR	-0.10	0.04	0.17	0.98
	LFP pre-STN	SPK VMNR	4.70	4.84	4.98	**$2.07 * 10^{-08}$**
	SPK pre-STN	LFP DLOR	-5.94	-5.80	-5.66	**$2.07 * 10^{-08}$**
	SPK pre-STN	SPK DLOR	-1.67	-1.54	-1.40	**$2.07 * 10^{-08}$**
	SPK pre-STN	LFP VMNR	-5.98	-5.84	-5.70	**$2.07 * 10^{-08}$**
	SPK pre-STN	SPK VMNR	-1.17	-1.03	-0.89	**$2.07 * 10^{-08}$**
	LFP DLOR	SPK DLOR	4.13	4.27	4.40	**$2.07 * 10^{-08}$**
	LFP DLOR	LFP VMNR	-0.18	-0.04	0.10	0.97
	LFP DLOR	SPK VMNR	4.63	4.77	4.91	**$2.07 * 10^{-08}$**
	SPK DLOR	LFP VMNR	-4.44	-4.30	-4.17	**$2.07 * 10^{-08}$**
	SPK DLOR	SPK VMNR	0.36	0.50	0.64	**$2.07 * 10^{-08}$**
	LFP VMNR	SPK VMNR	4.67	4.81	4.95	**$2.07 * 10^{-08}$**
**LFP vs SPK Exponent**	LFP pre-STN	SPK pre-STN	2.18	2.25	2.32	**$2.07 * 10^{-08}$**
	LFP pre-STN	LFP DLOR	0.11	0.19	0.25	**$2.07 * 10^{-08}$**
	LFP pre-STN	SPK DLOR	1.97	2.04	2.11	**$2.07 * 10^{-08}$**
	LFP pre-STN	LFP VMNR	0.07	0.14	0.21	**$4.56 * 10^{-07}$**
	LFP pre-STN	SPK VMNR	2.25	2.32	2.39	**$2.07 * 10^{-08}$**
	SPK pre-STN	LFP DLOR	-2.14	-2.07	-2.00	**$2.07 * 10^{-08}$**
	SPK pre-STN	SPK DLOR	-0.29	-0.21	-0.14	**$2.07 * 10^{-08}$**
	SPK pre-STN	LFP VMNR	-2.19	-2.12	-2.05	**$2.07 * 10^{-08}$**
	SPK pre-STN	SPK VMNR	0.00	0.07	0.14	0.07
	LFP DLOR	SPK DLOR	1.79	1.86	1.93	**$2.07 * 10^{-08}$**
	LFP DLOR	LFP VMNR	-0.12	-0.05	0.03	0.44
	LFP DLOR	SPK VMNR	2.07	2.14	2.21	**$2.07 * 10^{-08}$**
	SPK DLOR	LFP VMNR	-1.97	-1.90	-1.83	**$2.07 * 10^{-08}$**
	SPK DLOR	SPK VMNR	0.21	0.28	0.35	**$2.07 * 10^{-08}$**
	LFP VMNR	SPK VMNR	2.11	2.18	2.25	**$2.07 * 10^{-08}$**

Mean (Group 1) – mean (Group 2): the mean of Group 1 minus the mean of Group 2 is estimated. **Lower Limit**: the lower limit of the 95% confidence interval for the true difference of the means. **Upper Limit**: the upper limit of the 95% confidence interval for the true difference of the means. DLOR：dorsal lateral oscillatory region (STN motor domain). VMNR: ventral medial non-oscillatory region (STN non-motor domain). STN: subthalamic nucleus. Related to Figure 2.

Supplementary Table 3: The statistical analysis (N-way ANOVA) between non-rectified LFP and rectified LFP						
**Non-rectified LFP vs Rectified LFP Offset**	**Source**	**SS**	**DF**	**MS**	**F**	**Prob > F**
	Signal type	250.00	1	250.00	439.33	$\mathbf{1.17 * 10^{-87}}$
	Subregions	1.96	2	0.98	1.72	0.18
	Signal type*Subregions	0.02	2	0.01	0.02	0.98
	Error	1048.19	1842	0.57		
	Total	1300.17	1847			
**Non-rectified LFP vs Rectified LFP Exponent**	**Source**	**SS**	**DF**	**MS**	**F**	**Prob>F**
	Signal type	82.36	1	82.36	503.34	$\mathbf{9.40 * 10^{-99}}$
	Subregions	10.47	2	5.23	31.99	$\mathbf{2.21 * 10^{-14}}$
	Signal type*Subregions	0.032	2	0.016	0.098	0.91
	Error	301.38	1842	0.16		
	Total	394.24	1847			

Signal type indicates statistical analysis of offset (exponent) between LFP and spiking activity. **Subregions** indicates the statistical analysis of offset (exponent) between pre-STN, DLOR and VMNR. **Signal type*Subregions** represents the difference of offset (exponent) between the interaction effect of the signal types and sub-regions. SS: sum of squares due to each source. DF: degree of freedom associated with each source. MS: mean squares for each source. F: F-statistic. Prob > F: the p-value, which is the probability that the F-statistic can take a value larger than a computed test-statistic value. Related to Supplementary Figure 5

Supplementary Table 4: The multi-comparison of aperiodic parameters between non-rectified (Non-Rtf) LFP and rectified LFP in the three subregions of STN

Parameters	Group 1	Group 2	Lower Limit	Mean (Group 1) - mean (Group 2)	Upper Limit	P value
**Non-Rtf LFP vs Rectified LFP Offset**	Non-Rtf LFP pre-STN	Rectified LFP pre-STN	0.56	0.73	0.91	**$2.07 * 10^{-08}$**
	Non-Rtf LFP pre-STN	Non-Rtf LFP DLOR	-0.10	0.07	0.25	0.83
	Non-Rtf LFP pre-STN	Rectified LFP DLOR	0.65	0.82	0.99	**$2.07 * 10^{-08}$**
	Non-Rtf LFP pre-STN	Non-Rtf LFP VMNR	-0.14	0.04	0.21	0.99
	Non-Rtf LFP pre-STN	Rectified LFP VMNR	0.59	0.76	0.94	**$2.07 * 10^{-08}$**
	Rectified LFP pre-STN	Non-Rtf LFP DLOR	-0.83	-0.66	-0.49	**$2.07 * 10^{-08}$**
	Rectified LFP pre-STN	Rectified LFP DLOR	-0.09	0.08	0.27	0.73
	Rectified LFP pre-STN	Non-Rtf LFP VMNR	-0.87	-0.70	-0.53	**$2.07 * 10^{-08}$**
	Rectified LFP pre-STN	Rectified LFP VMNR	-0.14	0.03	0.20	1.00
	Non-Rtf LFP DLOR	Rectified LFP DLOR	0.57	0.74	0.92	**$2.07 * 10^{-08}$**
	Non-Rtf LFP DLOR	Non-Rtf LFP VMNR	-0.21	-0.04	0.13	0.99
	Non-Rtf LFP DLOR	Rectified LFP VMNR	0.52	0.69	0.86	**$2.07 * 10^{-08}$**
	Rectified LFP DLOR	Non-Rtf LFP VMNR	-0.96	-0.78	-0.61	**$2.07 * 10^{-08}$**
	Rectified LFP DLOR	Rectified LFP VMNR	-0.23	-0.06	0.12	0.95
	Non-Rtf LFP VMNR	Rectified LFP VMNR	0.56	0.73	0.90	**$2.07 * 10^{-08}$**
**Non-Rtf LFP vs Rectified LFP Exponent**	Non-Rtf LFP pre-STN	Rectified LFP pre-STN	0.34	0.43	0.52	**$2.07 * 10^{-08}$**
	Non-Rtf LFP pre-STN	Non-Rtf LFP DLOR	0.09	0.189	0.28	**$3.42 * 10^{-07}$**
	Non-Rtf LFP pre-STN	Rectified LFP DLOR	0.51	0.61	0.70	**$2.07 * 10^{-08}$**
	Non-Rtf LFP pre-STN	Non-Rtf LFP VMNR	0.04	0.14	0.23	**$3.75 * 10^{-04}$**
	Non-Rtf LFP pre-STN	Rectified LFP VMNR	0.46	0.55	0.64	**$2.07 * 10^{-08}$**
	Rectified LFP pre-STN	Non-Rtf LFP DLOR	-0.34	-0.25	-0.16	**$2.07 * 10^{-08}$**
	Rectified LFP pre-STN	Rectified LFP DLOR	0.08	0.18	0.27	**$1.03 * 10^{-06}$**
	Rectified LFP pre-STN	Non-Rtf LFP VMNR	-0.39	-0.29	-0.20	**$2.07 * 10^{-08}$**
	Rectified LFP pre-STN	Rectified LFP VMNR	0.02	0.12	0.21	**$4.43 * 10^{-03}$**
	Non-Rtf LFP DLOR	Rectified LFP DLOR	0.33	0.42	0.52	**$2.07 * 10^{-08}$**
	Non-Rtf LFP DLOR	Non-Rtf LFP VMNR	-0.14	-0.05	0.05	0.73
	Non-Rtf LFP DLOR	Rectified LFP VMNR	0.27	0.37	0.46	**$2.07 * 10^{-08}$**
	Rectified LFP DLOR	Non-Rtf LFP VMNR	-0.56	-0.47	-0.38	**$2.07 * 10^{-08}$**
	Rectified LFP DLOR	Rectified LFP VMNR	-0.15	-0.06	0.03	0.46
	Non-Rtf LFP VMNR	Rectified LFP VMNR	0.32	0.41	0.50	**$2.07 * 10^{-08}$**

Mean (Group 1) – mean (Group 2): the mean of Group 1 minus the mean of Group 2 is estimated. **Lower Limit**: the lower limit of the 95% confidence interval for the true difference of the means. **Upper Limit**: the upper limit of the 95% confidence interval for the true difference of the means. DLOR： dorsal lateral oscillatory region (STN motor domain). VMNR: ventral medial non-oscillatory region (STN non-motor domain). STN: subthalamic nuclear. **Non-Rtf:** non-rectified. Related to Supplementary Figure 5.
